# Turning Microstructure in Block Copolymer Membranes: A Facile Strategy to Improve CO_2_ Separation Performance

**DOI:** 10.1002/advs.202501330

**Published:** 2025-04-17

**Authors:** Jing Wei, Min Deng, Zikang Qin, Weiyi Zhao, Yujie Li, Roman Selyanchyn, Hongyong Zhao, Jie Dong, Dengguo Yin, Yuanfa Zhuang, Liyuan Deng, Lin Yang, Lu Yao, Wenju Jiang, Junfeng Zheng, Bart Van der Bruggen, Zhongde Dai

**Affiliations:** ^1^ College of Architecture and Environment Sichuan University Chengdu 610065 P. R. China; ^2^ National Engineering Research Centre for Flue Gas Desulfurization Chengdu 610065 P. R. China; ^3^ Carbon Neutral Technology Innovation Center of Sichuan Chengdu 610065 P. R. China; ^4^ College of Carbon Neutrality Future Technology Sichuan University Chengdu 610065 P. R. China; ^5^ National Synchrotron Radiation Laboratory University of Science and Technology of China Hefei 230029 P. R. China; ^6^ Imperial College London Exhibition Rd, South Kensington London SW7 UK; ^7^ Platform for Inter‐/Transdisciplinary Energy Research (Q‐PIT) Kyushu University 744, Motooka Nishi‐ku Fukuoka 819‐0395 Japan; ^8^ State Key Laboratory of Separation Membranes and Membrane Processes/National Center for International Joint Research on Separation Membranes Tiangong University Tianjin 300387 P. R. China; ^9^ School of Chemical Engineering and Technology Tiangong University Tianjin 300387 P. R. China; ^10^ DongFang Boiler Co., Ltd. Zigong 643001 P. R. China; ^11^ Clean Combustion and Flue Gas Purification Key Laboratory of Sichuan Province Deyang 618000 P. R. China; ^12^ Department of Chemical Engineering Norwegian University of Science and Technology Trondheim 7491 Norway; ^13^ Department of Chemical Engineering KU Leuven Celestijnenlaan 200F Leuven 3001 Belgium

**Keywords:** block copolymers, CO_2_ capture, membrane gas separation, microstructure rearrangement

## Abstract

To mitigate global climate change, the development of membranes with high CO_2_ permeability and selectivity is urgently needed. Here, a simple and effective non‐solvent‐induced microstructure rearrangement (MSR) technique is proposed to enhance the gas separation performance of Pebax 2533 membranes. By immersing Pebax 2533 membranes in amino acid salt solutions to induce MSR, the CO_2_ permeability of the optimized Pebax 2533‐GlyK 10 wt.% membrane reached 1180 Barrer, a 4.5‐fold increase compared to the original membrane, without compromising CO_2_/N_2_ selectivity. Moreover, the MSR membrane maintains stable gas separation performance for nearly 500 days, demonstrating excellent long‐term stability. Furthermore, applying the MSR technique to thin‐film composite (TFC) membranes revealed that both Pebax 2533/polyvinyl chloride (PVC) hollow fiber (HF) TFC membranes and Pebax 2533/polyacrylonitrile (PAN) flat‐sheet TFC membranes exhibited significantly enhanced CO_2_ permeance under the treatment of DI water. Characterization results indicated that the chemical‐physical properties of the membranes before and after MSR are nearly unchanged, suggesting that the non‐solvent‐induced MSR is a promising technique for next‐generation membrane development for carbon capture.

## Graphical abstract

A simple and effective non‐solvent‐induced microstructure rearrangement (MSR) technique is proposed to enhance the gas separation performance of Pebax 2533 membranes. By immersing Pebax 2533 membranes in amino acid salt solutions to induce MSR, the CO_2_ permeability of the Pebax 2533‐GlyK 10 wt.% membrane reached 1180 Barrer. Moreover, the MSR membrane maintains stable gas separation performance for nearly 500 days, demonstrating.

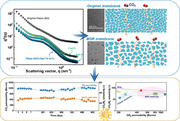

## 1 Introduction

Carbon capture, utilization, and storage (CCUS) have emerged as critical strategies to mitigate CO_2_ emissions with continued usage of fossil fuels.^[^ ^1^ ^]^ However, CO_2_ capture is, up to now, the most economically prohibitive stage in the CCUS process.^[^ ^2^, ^3^ ^]^ Within the available CO_2_ capture technologies, e.g., absorption, adsorption, chemical looping, and membrane separation, the latter has emerged as a rapidly developing technique with significant promise.^[^ ^4^, ^5^ ^]^ Its benefits encompass a compact footprint, better energy efficiency, and easier scalability.^[^ ^6^, ^7^, ^8^ ^]^

To enhance the commercial viability of the membrane‐based CO_2_ capture process, extensive research endeavors have been directed toward developing high‐performance CO_2_ separation membrane materials.^[^ ^9^, ^10^ ^]^ Turning the chemical structure of polymers, such as making the polymer backbones more rigid (e.g., polymers of intrinsic microporosity and their derivatives^[^ ^11^ ^]^) and/or introducing bulky side groups onto the polymer chain (e.g., polyimides (PIs),^[^ ^12^ ^]^ vinyl‐addition polynorbornenes,^[^ ^13^, ^14^ ^]^ Troger's based polymers^[^ ^15^ ^]^ and thermally arranged polymers (TR polymers)^[^ ^16^ ^]^) by complicated chemical structure design, has been proven to be an effective way to promote CO_2_ separation, especially CO_2_ permeability.^[^ ^17^ ^]^ All these polymer chains may pack less efficiently, creating more excess free volume for gas transport.^[^ ^18^ ^]^

On the other hand, turning the physical structure of polymeric membranes, especially block copolymer membranes, has also been proven to be a very effective method to control their morphology and pore size. This has been intensively studied for liquid separation,^[^ ^19^, ^20^ ^]^ but has been underexplored for gas separation.^[^ ^21^, ^22^ ^]^ It is well‐documented that the thermodynamic incompatibility between different blocks in block copolymers may lead to microphase separation during membrane formation,^[^ ^23^, ^24^ ^]^ resulting in various microstructures (e.g., spheres, layers, and cylinders) and, consequently, different mass transport characteristics.^[^ ^25^ ^]^ Thus, in principle, the CO_2_ transport properties of block copolymers can be turned by adequately controlling the membrane's physical microstructures.

In our previous research, the CO_2_ separation performance of several block copolymers has been enhanced by turning their microstructure by non‐solvent‐induced microstructure rearrangement (MSR). By using appropriate solvent‐nonsolvent combinations, the microstructure of the block copolymer‐based membrane was finely turned, transforming the random microstructure into a more organized one. This facilitated the formation of efficient CO_2_ transport pathways, thereby enhancing the CO_2_ separation efficiency within the membranes.^[^ ^26^, ^27^ ^]^ For instance, by incorporating the ionic liquid (IL) 1‐butyl‐3‐methylimidazolium tetrafluoroborate ([Bmim][BF_4_]) into the sulfonated pentablock copolymer Nexar, the microstructure can evolve from lamellar to cylindrical. The continuous cylindrical ionic domains facilitate CO_2_ transport and improve permeability. In another study, the same IL ([Bmim][BF_4_]) was introduced into Nafion and also served as a non‐solvent inducing an MSR, in which a better CO_2_ separation performance was obtained.^[^ ^28^ ^]^

Although ILs and polyethylene glycol (PEG) have been reported to be effective non‐solvents to induce MSR,^[^ ^28^, ^29^ ^]^ they may gradually leak out from the membrane, reducing the CO_2_ separation performance over time. Therefore, more recently, attempts have been made to employ water as a non‐solvent to turn the microstructure into polymeric membranes.^[^ ^26^, ^27^, ^30^ ^]^ For Nexar, Nafion, and Pebax 2533, using water as a non‐solvent to induce MSR has led to the formation of connected hydrophilic pathways, which is favorable for CO_2_ transport. For Nafion membranes, soaking in deionized (DI) water over 24 h resulted in a 2.3‐fold enhancement in CO_2_/N_2_ selectivity.^[^ ^30^ ^]^ In the case of the Pebax 2533 membranes, incubation in DI water for 24 h increased the CO_2_ permeability from 258.5 Barrer to 782.3 Barrer, with almost unchanged CO_2_/N_2_ selectivity. In addition, the corresponding morphological transformation was permanent unless the membrane was redissolved and re‐casted; the MSR Pebax 2533 demonstrated a stable permeability in 300‐day tests. All these results suggest that water can be an effective non‐solvent to induce MSR, leading to enhanced CO_2_ separation performance in block copolymer‐based membranes.

Inspired by the previous studies, in the current work, different types of amino acid salt solutions (glycine potassium salt (GlyK), proline potassium salt (ProK), and arginine potassium salt (ArgK)) were chosen as non‐solvents to induce MSR in Pebax 2533 membranes. To render the rearrangement, Pebax 2533 membranes were immersed in various non‐solvents for 24 h under ambient conditions. Hollow fiber (HF) MSR membrane modules were also developed. It was demonstrated that the Pebax 2533‐GlyK 10 wt.% membrane exhibited the highest CO_2_ permeability (1180 Barrer), which was a 4.5‐fold enhancement than the original Pebax membrane. Furthermore, the Pebax 2533‐GlyK membrane demonstrated nearly 500 days of long‐term stability, indicating substantial industrial application potential. Similarly, the MSR technique was proven effective in enhancing the performance of HF thin‐film‐composite (TFC) membranes. Thus, the utilization of non‐solvents to fabricate MSR membranes represents a simple and environmentally friendly strategy for developing CO_2_ separation membranes.

## 2 Results

### 2.1 Effect of Different Non‐Solvents on CO_2_ Separation Performance

**Figure 1:**
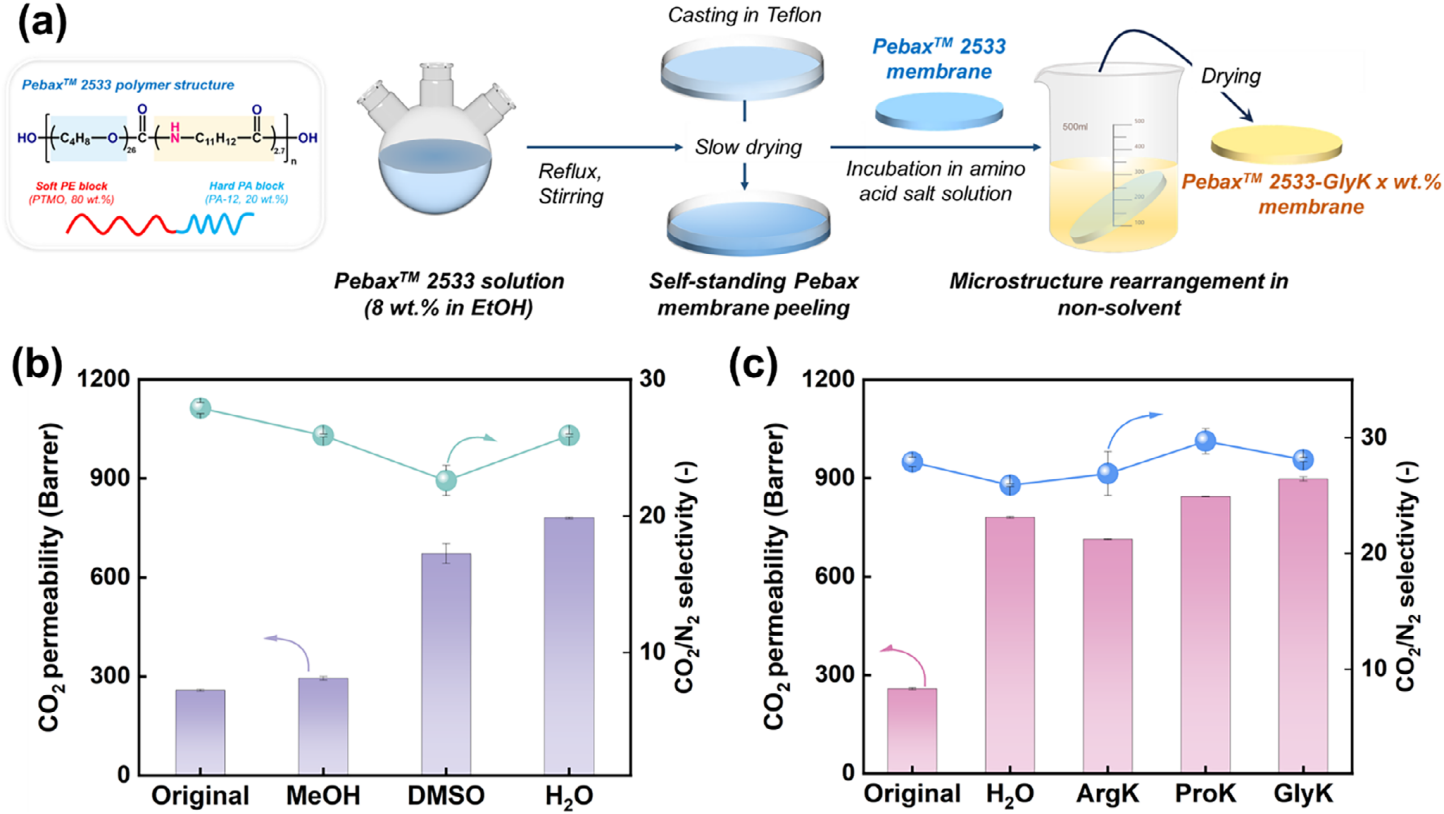
Gas separation performance of Pebax 2533 membranes before and after non‐solvents‐induced MSR. a) Schematic illustration of non‐solvent induced MSR fabrication protocol. Comparative gas transport properties of membranes treated with: b) DI water and organic solvents, and c) aqueous 2 wt.% amino acid salt solutions. All permeation experiments were conducted at 25 °C with a feed pressure of 2 bar.

The membrane fabrication protocol is schematically illustrated in **Figure** **1**a. To systematically evaluate the effects of non‐solvents on the gas separation performance of MSR membranes, in previous studies, DI water, methanol, and dimethyl sulfoxide were selected as non‐solvents to induce MSR in Pebax 2533 membranes. The test results showed that the CO_2_ permeability of the water‐treated membrane increased from 258.5 Barrer to 782.3 Barrer (≈3.0‐fold improvement). Meanwhile, the CO_2_/N_2_ selectivity was maintained (27.9 vs 25.9, as shown in Figure 1b).^[^ ^26^ ^]^

Building upon this foundation, various salt solutions were selected as non‐solvents to prepare Pebax 2533 MSR membranes. Initially, three different amino acid salt solutions (each at a concentration of 2 wt.%) were chosen as non‐solvents to induce MSR. The gas separation performance is shown in Figure 1c. Compared to membranes treated with DI water, membranes treated with amino acid salt solutions exhibited a higher CO_2_ permeability, with the GlyK‐treated membrane demonstrating the highest CO_2_ permeability (897.7 Barrer). Additionally, the CO_2_/N_2_ selectivity remains unchanged. On the other hand, improvements can also be found for membranes treated with two other amino acid salt solutions (i.e., AgrK and ProK), they also resulted in comparable CO_2_ permeabilities (ProK, 844.4 Barrer, ArgK, 713.9 Barrer). The observed variations in the effects of three amino acid salt solutions on the gas separation performance primarily stem from disparities in their chemical structures. Glycine is the structurally simplest amino acid containing merely a hydrogen atom in its side chain, facilitating homogeneous dispersion of its potassium salt (GlyK) within the polymeric matrix. This uniform distribution promotes the formation of continuous CO_2_ transport channels, thereby enhancing the effective diffusion of gases. In contrast, arginine has a larger molecular volume, which impedes the movement of the polymer's segments. Consequently, arginine forms aggregate through intermolecular interactions, disrupting the membrane's uniformity and reducing the effective gas permeation pathways. Furthermore, proline demonstrates intermediate behavior dictated by its unique cyclic configuration. The pyrrolidine ring in its side chain imposes restricted conformational mobility that prevents excessive chain rearrangement while maintaining moderate free‐volume distribution.

**Figure 2:**
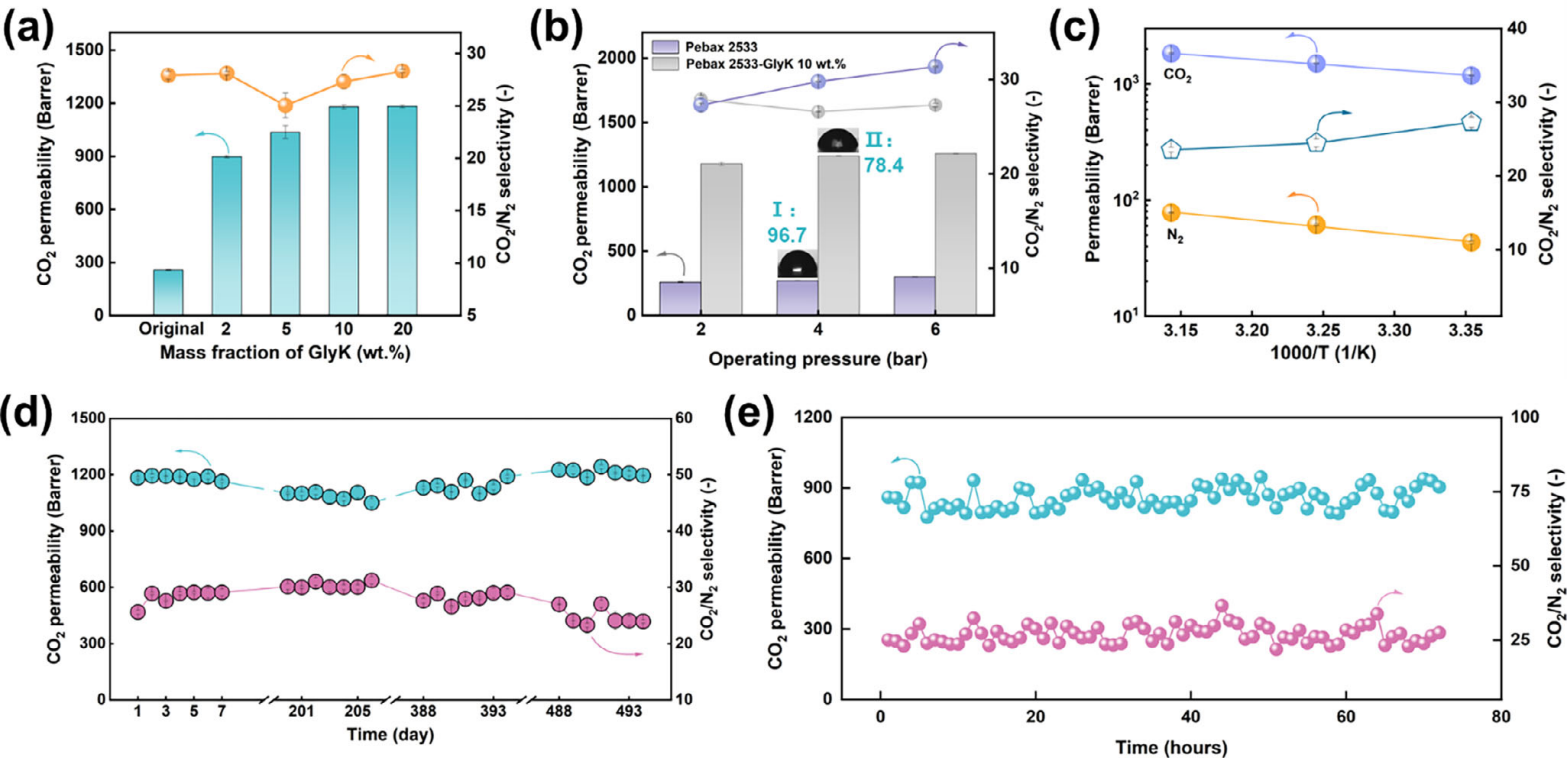
Gas separation performance of MSR membranes under different test conditions. a) CO_2_/N_2_ separation performance of Pebax 2533 membranes treated with varying concentrations of GlyK (single gas, 2 bar, and 25 °C). b) Effect of feed pressure on Pebax 2533 and Pebax 2533‐GlyK 10 wt.% membrane gas separation performance (Single gas, 25 °C) (The illustration depicts the water contact angles of the Pebax 2533 membrane before and after MSR process: I. Original membrane; II. MSR membrane treated in 10% GlyK solution). c) Effect of feed temperature on Pebax 2533‐GlyK 10 wt.% membrane gas separation performance (single gas, 2 bar). d) Long‐term stability testing of Pebax 2533‐GlyK 10 wt.% membrane in single gas permeation tests (e), and stability in mixed gas permeation tests (feed pressure 2 bar, 25 °C).

Attempts have been made to optimize the amino acid solution concentration, and the results are shown in Figures S12 and S15 (Supporting Information). It was found that an increasing ProK concentration reduced the CO_2_ permeability while an increasing ArgK concentration led to a moderate fluctuation in CO_2_ permeability. Interestingly, from **Figure** **2**a, increasing the GlyK concentration to 20 wt.% resulted in a much higher CO_2_ permeability (1182 Barrer), and the overall CO_2_ separation performance is much better than the other two amino acid salts. Therefore, the superior‐performing Pebax 2533‐GlyK membranes were selected for further gas separation testing and characterization.

### 2.2 Self‐Standing Membranes

The effect of GlyK concentration on the CO_2_ separation performances was systematically evaluated, as presented in Figure 2a. Remarkably, when the GlyK concentration was increased from 2 to 10 wt.%, the CO_2_ permeability improved from 897.7 Barrer to 1179.5 Barrer, which is 4.5 times that of the original Pebax 2533 membrane ($P_{CO_{2}}$ ≈ 200 Barrer,^[^ ^31^, ^32^ ^]^ while $\alpha_{CO_{2}/N_{2}}$ ≈ 28). Even though further increasing the GlyK concentration to 20 wt.% resulted in a slight improvement in CO_2_ permeability (1182.6 Barrer), considering the other practical issues and costs, 10 wt.% was considered the optimal GlyK concentration. Considering the $\alpha_{CO_{2}/N_{2}}$, it remains almost unchanged before and after the MSR process (25–30). Again, this possibly suggests that the GlyK non‐solvent induced MSR process is mainly a physical process, as the CO_2_/N_2_ selectivity was dominated by the intrinsic CO_2_‐philic domains in the Pebax 2533 matrix.

Extensive studies have established that the feed gas temperature and pressure critically influence on both CO_2_ permeability and CO_2_/N_2_ selectivity.^[^ ^33^ ^]^ Consequently, the effects of feed pressure and temperature on Pebax membranes obtained in this study were also studied (Figure 2b,c). As indicated in the figure, an increase in pressure from 2 to 6 bar leads to a gradual rise in the CO_2_ permeability of the membrane, aligning well with reported values in the literature.^[^ ^34^, ^35^ ^]^ Meanwhile, the CO_2_/N_2_ selectivity maintains relative stability, underscoring that the Pebax 2533‐GlyK membrane shares comparable CO_2_ separation behavior with the original Pebax 2533 membrane. Similar to other CO_2_‐selective membranes, elevating the test temperature leads to a higher CO_2_ permeability at the expense of reduced CO_2_/N_2_ selectivity.^[^ ^36^ ^]^ In this study, changing the test temperature from 25 °C to 45 °C led to a sharp increase in $P_{CO_{2}}$ (from 1179.5 to 1829.7 Barrer) and a moderate decline in $\alpha_{CO_{2}/N_{2}}$ (from 27.3 to 23.6) for Pebax 2533‐GlyK 10 wt.% membranes.

In addition, for a given polymer, the influence of temperature on gas permeability (P) can be elucidated using the Arrhenius equation:^[^ ^37^ ^]^

(1) $$P=P_{0}\;.exp\left(-\frac{E_{p}}{RT}\right)$$

where *E_p_* and *P_0_* represent activation energy and pre‐exponential factor, respectively, *R* is the molar gas constant, and *T* stands for temperature. In this study, for the Pebax 2533‐GlyK 10 wt.% membrane, the *E_p_* values were 17.3 and 23.1 kJ mol^−1^, for CO_2_ and N_2_, respectively. Extracted *E_p_* values are summarized in Table S3 (Supporting Information). Analogous to other polymeric membranes (e.g., polyether, Nafion, and PI), the activation energy of CO_2_ consistently remains below that of N_2_.

Long‐term operational stability constitutes a critical performance metric for practical gas separation membranes. Pebax 2533‐GlyK 10 wt.% was selected for durability test under both single and mixed gas conditions with a feed pressure of 2 bar at 25 °C. In the single gas permeation test, the membrane was tested continuously for 7 days, then stored in ambient conditions for 200 days and ≈500 days, and then tested again. It was found that after 200 days, 400 days, and 500 days of aging, the $P_{CO_{2}}$ (from 1160 to 1090 Barrer) and $\alpha_{CO_{2}/N_{2}}\;$(from 26 to 30) remained almost unchanged (Figure 2d). Furthermore, mixed gas (10:90 CO_2_/N_2_) permeation tests were also tested for 72 h, the membrane also exhibited excellent long‐term stability in both $P_{CO_{2}}$ (859.0 Barrer) and $\alpha_{CO_{2}/N_{2}}$ (27) (Figure 2e). The excellent long‐term stability makes the Pebax 2533‐GlyK‐10% membrane a promising candidate for practical applications.^[^ ^31^, ^38^ ^]^

### 2.3 Chemical and Physical Characterization

**Figure 3:**
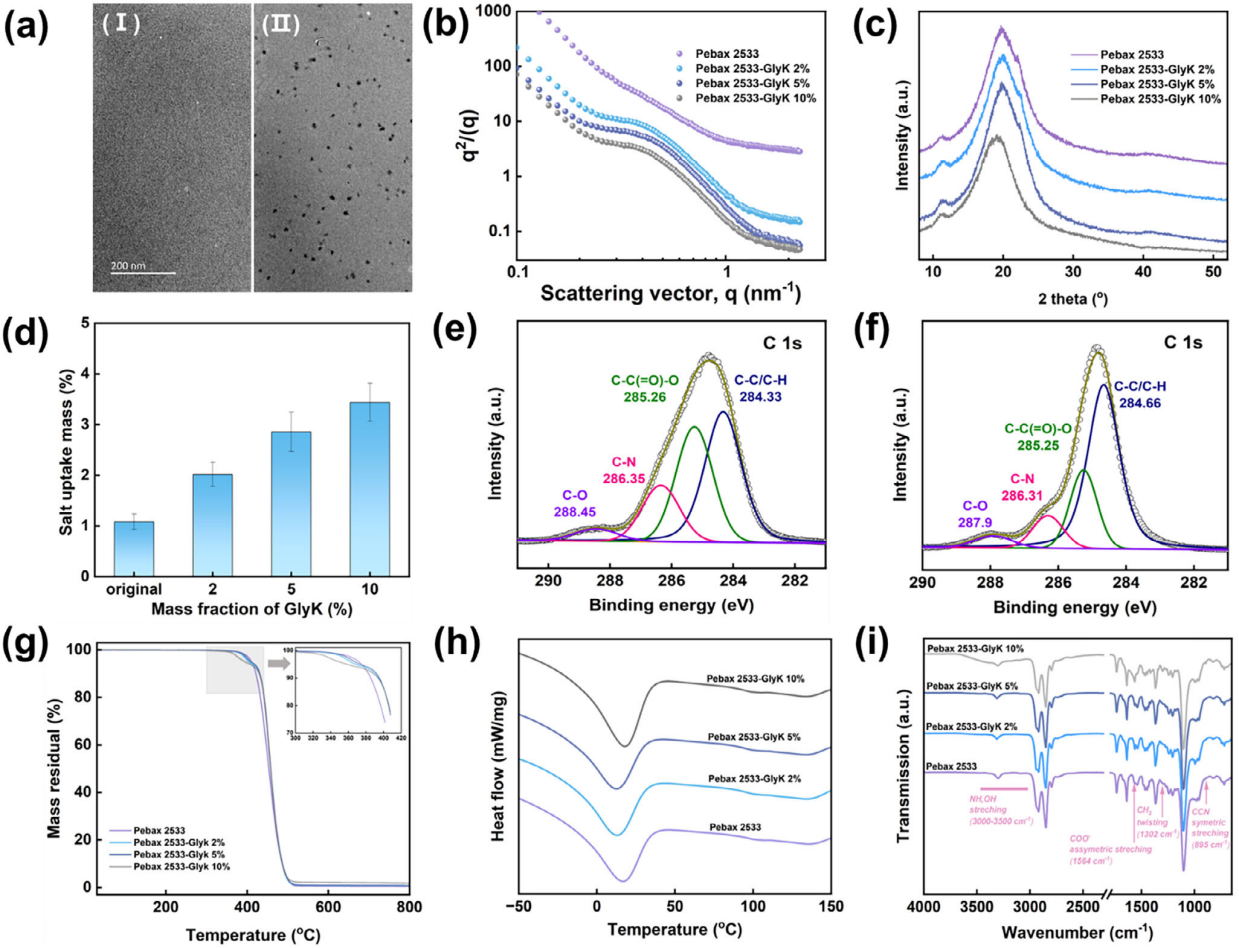
Physical and chemical characterization of Pebax 2533 membrane before and after MSR. a) TEM images of original Pebax 2533 membrane (I) and Pebax 2533 membrane rearranged in 10% GlyK solution (II). b) SAXS and c) XRD spectra of Pebax 2533 membrane before and after MSR in GlyK solution. d) Salt uptake of Pebax 2533 membrane in GlyK solution. e) Narrow XPS C 1s spectrum of the original Pebax 2533 and f) Pebax 2533‐GlyK 10% membranes. g) TGA results, h) DSC analysis, and i) FTIR spectra of Pebax 2533 membrane before and after MSR in GlyK solution.

The microstructure of the original Pebax 2533 and Pebax 2533‐GlyK 10 wt.% membranes was examined using transmission electron microscopy (TEM). The images revealed dark regions corresponding to ion‐rich hydrophilic domains (polytetramethylene oxide (PTMO)) stained with saturated lead acetate, and light regions representing unstained hydrophobic regions (polyamide (PA)).^[^ ^26^ ^]^ In addition, as shown in **Figure** **3**a **(II)**, MSR in the membrane treated in GlyK solution leads to a larger size of the PTMO domains (in good agreement with the small‐angle X‐ray scattering (SAXS) results presented in Figure 3b), these domains may be the evidence of a well interconnected PTMO network formed in the membrane after the incubation in the non‐solvent, which is favorable for CO_2_ transport. Furthermore, interestingly, GlyK salt nanodomains within the membranes with sizes of less than 10 nm with even distribution were also observed. The presence of these salt domains may positively improve CO_2_ transport in the MSR membranes.

The free volume and defects of the membranes before and after MSR were investigated using positron annihilation lifetime spectroscopy (PALS). Analysis of the PALS data (Table S1, Supporting Information) indicates that τ_1_ and τ_2_ increase in the MSR membranes, suggesting that immersion in a nonsolvent leads to an increase in both small and large free volumes within the membranes. This expansion provides additional gas diffusion pathways, thereby significantly enhancing the CO_2_ permeability. Moreover, the parameters τ_3_, I_3_, R, and FFV show a minimal variation between the two samples, indicating that MSR does not significantly alter the maximum free volume. This stability likely aids in maintaining the material's mechanical strength and structural integrity.

It is reported that the CO_2_ transport in block copolymers is closely related to the dimensions, configuration, and interconnectivity of hydrophilic domains.^[^ ^39^ ^]^ SAXS spectrum reveals a faint peak ≈*q* = 0.4 nm^−1^ for the original Pebax 2533 membrane (Figure 3b), corresponding to a characteristic spacing (d) of ≈15.7 nm, this characteristic interval is associated with the hydrophilic domains, aligning with observations made by Wilkes and others.^[^ ^40^ ^]^ Upon immersion in a GlyK solution, an increase in GlyK concentration results in the broadening of the peak and a shift toward lower q values in the SAXS spectrum. This change signifies that the corresponding spacing increases to ≈18.5 nm. This may have a positive impact on permeability. A similar trend has also been found in our previous reports.^[^ ^26^, ^41^ ^]^ Soaking Pebax 2533 in DI water also resulted in a significant MSR, resulting in an increase of CO_2_ permeability by over three times.^[^ ^26^ ^]^ In our previous study, introducing IL (i.e., [Bmim][BF_4_]) into Nexar as a non‐solvent also caused a significant structural transformation from a lamellar structure to a layered structure,^[^ ^41^ ^]^ leading to a substantial enhancement in CO_2_ permeability.

In X‐ray diffraction (XRD) curves, an intense crystalline peak was observed at 20.0° for the original Pebax 2533 membrane, attributed to the PA crystalline region (Figure 3c), in alignment with published literature.^[^ ^42^ ^]^ Interestingly, membrane immersion in GlyK solution exhibited a gradual decline in peak intensity, possibly attributed to the hydrogen bond rupture within the PA segments, leading to a reduction in the binding energy and subsequent MSR.^[^ ^43^ ^]^ The salt uptake of the membrane was also measured and results are shown in Figure 3d. As the GlyK concentration increased in the solution, the salt uptake of the Pebax 2533 membrane gradually increased.^[^ ^26^, ^41^ ^]^ The thermogravimetric analysis (TGA) results (Figure 3g) further confirmed the salt uptake, as the thermal decomposition of the MSR membranes differed slightly from that of the original polymer^[^ ^44^ ^]^ Interestingly, the observed trend in salt uptake in the membranes was consistent with the permeability results, namely that higher salt uptake leads to a higher CO_2_ permeability. Water contact angle (CA) measurement was carried out to investigate changes in hydrophilicity before and after MSR of the Pebax 2533 membranes (Figure 2b). It can be observed that the CA value for the original Pebax 2533 membrane is ≈96.7°, indicating its hydrophobic nature. After immersion in 10 wt.% GlyK solutions for 24 h, the CA decreased to ≈78.4°. This indicates that the Pebax 2533 membranes became hydrophilic after MSR in both non‐solvents.

X‐ray photoelectron spectroscopy (XPS) analysis was conducted to further elucidate the elemental composition of both the original and MSR membranes. The C1s spectra are shown in Figure 3e,f. The substantial increase in the C─C/C─H bond from 44.2 to 63.6% implies an increase in the proportion of C─C and C─H bonds, characteristic of the polymer backbone. This increase suggests that the physical interaction between Pebax 2533 and GlyK might have led to a reorganization or rearrangement of the polymer chains, possibly resulting in increased chain flexibility and/or mobility.

In addition, thermal analysis was also carried out, and the results are shown in Figure 3g. TGA results reveal that the rearrangement process has a negligible effect on the overall thermal stability, all the MSR membranes maintained the superior thermal stability of the original Pebax membranes, and almost no thermal degradation could be found below 400 °C, fulfilling the requirements for post‐combustion CO_2_ capture.^[^ ^45^ ^]^ The differential scanning calorimetry (DSC) results (Figure 3h) demonstrate that the glass transition temperature (*T_g_*) of membranes exhibits only minor variations following treatment with GlyK solutions at different concentrations, indicating preserved thermal stability in the MSR membranes, which was consistent with TGA. Furthermore, the negligible *T_g_* fluctuations suggest this parameter does not play a dominant role in governing gas separation performance. Combined with microstructural characterization (TEM and SAXS), these observations imply that the gas permeability improvement may stem from the rearrangement of block copolymer chain segments rather than conventional crystallinity evolution processes.

Fourier‐transform infrared spectroscopy (FTIR) was also employed to characterize the membranes, and the results are shown in Figure 3i. Compared to the original Pebax 2533, the chemical structure was almost unchanged for Pebax 2533 membranes after GlyK solution treatment. The intensity of the characteristic peaks of GlyK gradually increased as the GlyK concentration increased from 2 wt.% to 10 wt.%. Characteristic peaks were seen in Pebax 2533 GlyK‐10 wt.% (as indicated by red arrows in the figure), proving the presence of GlyK salt in the Pebax 2533 matrix. Combined with the negligible variation of *T_g_* shown by DSC, it is speculated that GlyK is likely distributed in the Pebax 2533 matrix through a physical mixture. Furthermore, no detectable alterations in characteristic peaks were observed in the membrane following MSR. Therefore, it is speculated that the MSR technique may be a physical process.^[^ ^30^ ^]^ In the supplementary information, a series of characterizations for the micro‐structure rearranged membranes prepared using ArgK solution as non‐solvents is presented. These are illustrated in Figures S6–S11 (Supporting Information) (Pebax‐ArgK).

### 2.4 Thin Film Composite Membrane Fabrication

TFC membranes are widely utilized in practical gas separation applications due to the thin selective layer, which facilitates a higher transmembrane gas flux.^[^ ^46^ ^]^ In this section, Pebax 2533 TFC membranes were made on both HF and flat sheet porous supports, and a non‐solvent‐induced MSR process was applied to these TFC membranes. In the current study, polyvinyl chloride (PVC) HF and polyacrylonitrile (PAN) flat sheet ultrafiltration membranes were selected as porous supports due to a combination of proper molecular weight cut‐off (≈20 kDa) and reasonable flatness of the membrane surfaces.

**Figure 4:**
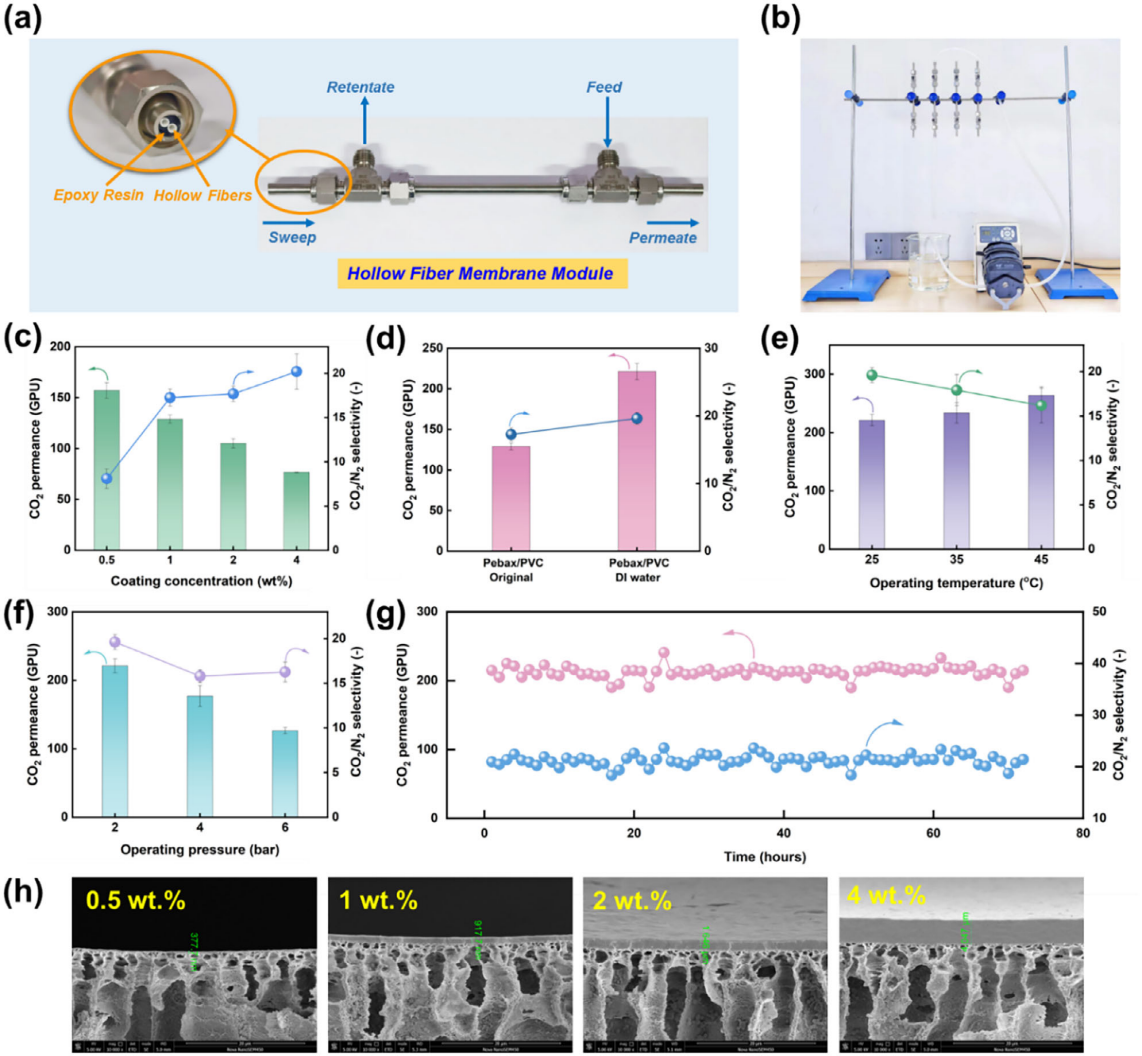
a) Optical images of the HF membrane modules and b) HF membrane module coating set‐up. c) Impact of coating solution concentration on CO_2_/N_2_ separation performance (25 °C, 2 bar). d) CO_2_/N_2_ separation performance of HF membrane modules before and after MSR (25 °C, 2 bar). Impact of test temperature (e) and feed pressure (f) on CO_2_/N_2_ separation performance of PVC HF membranes. g) Long‐term stability testing of HF Pebax/PVC TFC membranes (25 °C, 2 bar). h) SEM cross‐sectional images of the HF TFC membranes with varying casting solution concentrations. All gas permeation tests were conducted under mixed gas conditions with a CO_2_/N_2_ ratio of 10/90 (vol%).

In addition, instead of the traditional dip‐coating method widely used for TFC membrane fabrication, a facile in situ HF membrane coating method was applied to coat the Pebax selective layer onto the PVC HF support. **Figure** **4**a,b illustrate the PVC HF membrane modules and the in‐situ membrane coating set‐up, respectively. HF Pebax/PVC TFC membranes were prepared by pumping the Pebax 2533 solution through the core side of the HF, the membrane thickness can be controlled by the coating solution concentration and the number of coating cycles. As illustrated in Figure 4c, at a casting solution concentration of 0.5 wt.%, the CO_2_ permeance of the membrane module was 157.1 GPU, as the concentration increased to 4 wt.%, the CO_2_ permeance decreased to 76.80 GPU, while the CO_2_/N_2_ selectivity rose from 8.1 (0.5 wt.%) to 20.2 (4 wt.%). Scanning electron microscopy (SEM) observation of the membrane cross‐section (Figure 4h) revealed that the selective layer thickness increased from ≈380 nm to ≈4.0 µm, leading to increased mass transport resistance and resulting in a reduced CO_2_ permeance.

Following the approach of our previous study, the Pebax/PVC HF TFC membrane modules fabricated via 1 wt.% Pebax coating solution was immersed in DI water and 10 wt.% GlyK solution to induce MSR. Figure 4d shows that when DI water was used as the non‐solvent, the CO_2_ permeance increased from 128.9 GPU to 221.2 GPU, which was a 1.7‐fold enhancement, and the CO_2_/N_2_ selectivity also increased from 17.26 to 19.62. In contrast, when 10 wt.% GlyK solution was used as a non‐solvent, both CO_2_ permeance and CO_2_/N_2_ selectivity were significantly dropped, possibly due to the formation of GlyK crystals in the membrane pores that sharply increased the mass transport resistance (Figure S21, Supporting Information). Furthermore, a flat sheet PAN support was also applied to fabricate Pebax 2533 TFC membranes. As illustrated in Figure S22 (Supporting Information), after DI water treatment, both CO_2_ permeance and CO_2_/N_2_ selectivity were significantly improved, denoting the non‐solvent treatment is also working for TFC membranes.

The effect of feed temperature and feed pressure on the TFC membrane gas separation performance was also investigated; results are shown in Figure 4e,f. Similar to many solution‐diffusion‐based membranes,^[^ ^36^ ^]^ as the testing temperature increases, the CO_2_ permeance rises while the CO_2_/N_2_ selectivity decreases. In the case of feed pressure, as shown in Figure 4f, as the feed pressure increases, both CO_2_ permeance and CO_2_/N_2_ selectivity gradually decrease, possibly due to the competitive sorption and plasticization of the TFC membranes.

Durability assessments of the HF Pebax/PVC‐DI water and Pebax/PAN‐DI water TFC membranes were conducted under mixed gas conditions with a feed pressure of 2 bar at 25 °C. As illustrated in Figure 4g and Figure S23 (Supporting Information), both TFC membranes exhibited exceptional stability in CO_2_ permeability and CO_2_/N_2_ selectivity throughout the testing period. This remarkable long‐term stability substantiates the commercial viability of MSR TFC membranes for practical gas separation applications.

### 2.5 Comparison of Membrane CO_2_/N_2_ Separation Performance

**Figure 5:**
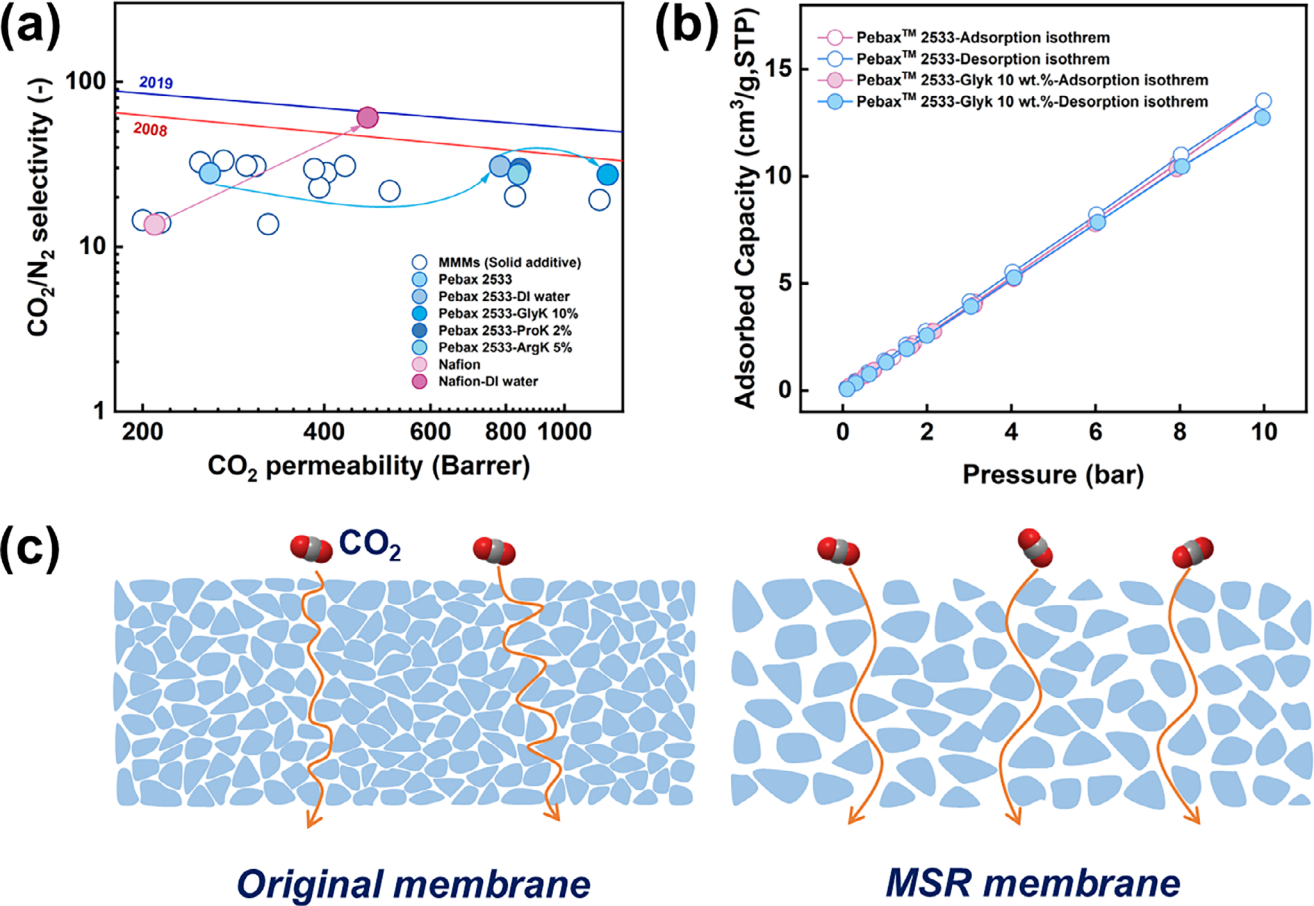
CO_2_/N_2_ separation performance comparison and possible mechanism of MSR in Pebax 2533 membranes. a) The CO_2_/N_2_ separation performance comparison in Robeson Upper Bound plot (detailed gas permeation data can be found in Table S4, Supporting Information). b) CO_2_ adsorption isotherms of original and MSR membrane. c) Gas transport mechanism in original and MSR membranes.

The CO_2_/N_2_ separation performance of membranes before and after the MSR process was plotted on the Robeson upper bound plot (**Figure** **5**a). Gas permeation data of Pebax 2533 and mixed matrix membranes based on the same polymer were also included (detailed gas permeation data shown in Table S4, Supporting Information). In our previous study, it was demonstrated that MSR can effectively promote the CO_2_/N_2_ separation performance for Nafion, Nexar, and Pebax 2533 membranes by soaking the membranes in DI water for 24 h.^[^ ^26^, ^30^ ^]^ For instance, the CO_2_ permeability of Pebax 2533‐DI water MSR membranes was ≈3‐fold higher than that of the original Pebax 2533 membranes.^[^ ^26^ ^]^ Furthermore, treating the membranes with 10 wt% GlyK solution resulted in an even higher CO_2_ permeability, which was ≈4.5‐fold higher than for the original Pebax 2533 membrane, while the CO_2_/N_2_ selectivity maintained (≈27), demonstrating the membranes are more competitive for CO_2_ separation applications. It has been reported that the CO_2_ permeability of Pebax membranes can also be improved by introducing either CO_2_‐philic liquids (e.g., IL or PEG)^[^ ^47^ ^]^ or porous nanofillers (e.g., metal organic frameworks into the Pebax 2533 matrix,^[^ ^48^ ^]^ these approaches either increase the membrane preparation complexity or lead to other problems like poor compatibility between the filler and Pebax matrix. Moreover, the long‐term stability of such membranes is still a problem. In contrast, the membranes fabricated via the MSR strategy avoid the above‐mentioned issues and demonstrate excellent long‐term stability. During gas permeability testing, the rapid transport rate of gases through the Pebax 2533 membrane precluded reliable evaluation of diffusion coefficient variations via the time‐lag method. We therefore characterized CO_2_ adsorption isotherms of the Pebax 2533 membrane, both before and after MSR. As demonstrated in Figure 5b, CO_2_ solubility remained virtually unchanged between the original and MSR membranes. According to the solution‐diffusion mechanism governing gas separation membranes, where permeability is determined by the product of solubility and diffusion coefficient, the almost unchanged CO_2_ solubility in the MSR membranes the improvement of the gas permeability mainly comes from the diffusivity improvement.^[^ ^49^, ^50^ ^]^

The possible gas transport mechanisms in original and MSR membranes are shown in Figure 5c. As noted earlier, in comparison to the original membrane, the MSR membrane exhibits only a negligible shift in *T_g_* (Figure 3h), suggesting that the variation in gas transport properties of the Pebax 2533 membrane is not governed by changes in the crystallinity of the membrane, but rather primarily regulated by alterations in its microstructure. Furthermore, SAXS analysis revealed that the characteristic spacing of the MSR membrane is greater than that of the original membrane (18.5 vs 15.7, as shown in Figure 3b), thereby effectively decreasing the tortuosity of the gas transport channels and significantly improving gas permeability.

## 3 Discussion

To improve the gas separation performance, a facile non‐solvent induced MSR approach has been developed to turn the physical structure of polymeric membranes. This innovative MSR strategy demonstrated remarkable efficacy in both self‐standing membranes and TFC membranes fabricated with Pebax 2533. Notably, self‐standing membranes treated with 10 wt.% GlyK solution achieved a CO₂ permeability of 1179.5 Barrer, representing a 4.5‐fold enhancement over original membrane. Furthermore, the MSR membranes also exhibited superior long‐term stability over nearly 500 days. In addition, the MSR method was also applied to TFC membranes fabricated on both HF and flat sheet porous support, in both cases, the CO_2_ permeance was significantly improved (from 128.9 GPU to 221.2 GPU for HF TFC membrane and from 324.0 GPU to 403.6 GPU for flat sheet TFC membrane) after MSR in DI water. Meanwhile, TGA, FTIR, and XPS results reveal that amino acid salt‐induced MSR is a physical process, the chemical‐physical properties of the MSR membrane are almost the same compared to the precursor.

Overall, the non‐solvents‐induced MSR method is an environmentally friendly and facile approach to enhance the gas separation performance of block copolymer membranes. Future work may be carried out by applying this method to other block copolymers, as well as applying this method to larger‐scale CO_2_ separation membrane modules (e.g., >10 m^2^).

## 4 Experimental Section

### Self‐Standing Membrane Fabrication

The self‐standing Pebax 2533 membrane was fabricated using a solution casting method, as described below: The casting solution was prepared by adding Pebax 2533 (8 g) and ethanol (92 g) into a single‐mouth flask, followed by reflux‐stirred at 80 °C for 8 h to gain a homogenous solution. Subsequently, a desired amount of the Pebax 2533 solution was poured into a teflon Petri dish and allowed to evaporate gradually at room temperature (RT) over 24 h. Afterward, the membrane was transferred to a vacuum oven and dried at 35 °C for 12 h to ensure the complete removal of residual solvent.

### Self‐Standing MSR Membrane

The amino acid salt solutions were prepared by dissolving three different amino acids (Glycine (Gly), Arginine (Arg), and Proline (Pro)) with KOH in DI water, resulting in solutions with mass fractions of 0, 2, 5, 10, and 20 wt.%, respectively. Pebax 2533 membranes were immersed in the non‐solvents for 24 h under RT conditions. The membranes were subsequently transferred to a vacuum oven, dried at 35 °C for 12 h, and then subjected to analysis.

### TFC Membrane Fabrication

The HF Pebax/PVC TFC membrane was prepared via the following steps: First, the commercial PVC HF membranes were washed with tap water for 6 h, followed by DI water for another 6 h, and then dried in a vacuum oven at 35 °C for 12 h. Subsequently, the PVC HF membranes were placed in the membrane module, with two fibers per module, and sealed with epoxy resin. The casting solutions of varying concentrations (diluting the 8 wt.% Pebax 2533 casting solution to 0.5, 1, 2, and 4 wt.%, respectively) were pumped from top to bottom into the membrane module using a peristaltic pump at a speed of 5 rpm, with a coating time of 10 s. After coating, the membrane module was placed vertically in a drying oven at 35 °C for 12 h to dry the casting solution.

The flat sheet Pebax/PAN TFC membrane was prepared via a dip‐coating method: The PAN/nonwoven fabric composite porous support layer was affixed to a glass plate with the surface outward and the back (nonwoven fabric side) inward. The glass plate with PAN/nonwoven fabric composite porous support was dipped into the casting solutions of different concentrations (dilute the 8 wt.% Pebax 2533 casting solution to 1, 2, and 4 wt.%, respectively) for ≈10 s, then withdrawn and subjected to drying at 35 °C for 1 h. Subsequently, the glass plate was inverted 180° to repeat the coating procedure. This process was repeated twice to ensure the uniform thickness of the selective layer. Finally, the Pebax/PAN TFC membranes were obtained by vacuum drying at 35 °C for 12 h.

### TFC Membrane MSR

For the non‐solvent‐induced MSR, DI water and a 10 wt.% GlyK solution were selected as non‐solvents. As shown in Figure S4 (Supporting Information), HF membrane modules, and flat sheet membrane materials were immersed in the non‐solvents under RT conditions for 24 h. Subsequently, the membranes were dried in a vacuum oven at 35 °C for 12 h before analysis.

Gas permeation measurements: The single gas permeability was assessed using the constant‐volume variable‐pressure technique,^[^ ^51^ ^]^ schematically shown in Figure S2 (Supporting Information). The gas permeability (*P*) was determined by Equation (2):

(2) $$P=\frac{V_{d}}{\mathrm{RTA}}\;\times \frac{l}{\left(p_{u}-p_{d}\right)}\times \left[{\left(\frac{\mathrm{dp}}{\mathrm{dt}}\right)}_{t\to \infty }-{\left(\frac{\mathrm{dp}}{\mathrm{dt}}\right)}_{\mathrm{leak}}\right]$$

where *V_d_* stands for the downstream volume, *R* is the universal gas constant, *T* represents the test temperature, *A* corresponds to the effective permeation area, *l* is the membrane thickness, and *p_u_‐p_d_* indicates the transmembrane pressure. Furthermore, ${\left(\frac{\mathrm{dp}}{\mathrm{dt}}\right)}_{t\to \infty }$ denotes the steady‐state pressure gradient on the downstream side, while ${\left(\frac{\mathrm{dp}}{\mathrm{dt}}\right)}_{\mathrm{leak}}$ represents the leak rate of the permeation rig that was measured for the whole rig after full evacuation. The membrane thickness was gauged using a digital caliper (San Liang, China), and an average of at least 10 measurements were recorded across the entire permeation region. The permeation area was measured using ImageJ software, and an average of at least ten measurements was recorded.

The ideal gas selectivity (α*) is described as the ratio of permeability for different gas components:

(3) $$\alpha_{\mathrm{ij}}^{*}=\frac{P_{i}}{P_{j}}$$

here, *P_i_* and *P_j_* represent the permeabilities of different gaseous species *i* and *j*, and the gas permeability was calculated in Barrer units (1 Barrer = 10^−10^(cm^3^(STP)·cm/(cm^2^·s·cmHg). The reported results for each sample represent the average of a minimum of three measurements.

The mixed gas permeability/gas permeance was measured using the GY‐4 Mixed Gas Permeation System (Nanjing Hober, China) as schematically shown in Figure S3 (Supporting Information). The feed gases consisted of CO_2_ and N_2_ in a 10/90 vol% ratio. The mass flow rates on both the feed and sweep sides were regulated using a mass flow controller, while a back‐pressure regulator was employed to maintain the feed‐side pressure. Gas chromatograph (GC 9790 Plus, Fuli Instruments, China) analyzed the gas compositions in both the permeate and retentate streams. During testing, the temperature and pressure of the feed gas were adjusted as required. The calculation formula for gas permeance (*Q_i_*) was determined by Equation (4):

(4) $$Q_{i}=\frac{N_{i}y_{i}L}{A\left(\langle p_{i,feed},p_{i,ret}\rangle -p_{i,perm}\right)}$$

In the above equation, *N_i_* is the total permeate flow, 𝑦_𝑖_ denotes the mole fraction of gas 𝑖 in the permeate. 𝐴 stands for the effective membrane area, *L* denotes the thickness of the membrane, while *p_i, feed_*, *p_i, ret,_* and *p_i, perm_* represent the partial pressures of the *i*th species in the feed, retentate, and permeate, respectively.

The formula for calculating gas selectivity is shown in Equation (5):

(5) $$\alpha_{ij}=\frac{y_{i}/y_{j}}{x_{i}/x_{j}}$$

In the above equation, 𝑦_𝑖_ and 𝑦_𝑗_ represent the molar ratios of gas 𝑖 and gas 𝑗 on the permeation side, respectively. *𝑥_𝑖_* and *𝑥_𝑗_* represent the molar ratios of gas 𝑖 and gas 𝑗 on the retentate side, respectively.

To minimize experimental error, the permeance of each gas for each membrane was tested at least three times, and all gas separation data included error bars.

## Conflict of Interest

The authors declare no conflict of interest.

## Author Contributions

Z.D.D., W.J.J., L.Y.D., and Y.F.Z. performed in conceptualization. J.W., Z.D.D., D.G.Y., L.Y., and L.Y. performed in methodology. J.W., D.M., Z.K.Q., W.Y.Z., Y.J.L., H.Y.Z., and J.D. performed in investigation. J.W. and R.S. performed in visualization. ZDD, WJJ, YFZ, JFZ Supervision. JW Writing original draft. Z.D.D., R.S., L.Y.D., and B.V.B. Writing: review and editing.

## Supporting information



Supporting Information

## Data Availability

The data that support the findings of this study are available from the corresponding author upon reasonable request.
