# Supplementary material for: Turning Microstructure in Block Copolymer Membranes: A Facile Strategy to Improve CO2 Separation Performance
Source: Adv Sci (Weinh). 2025 Apr 17;12(25):2501330. doi: 10.1002/advs.202501330 (PMC12224946; doi:10.1002/advs.202501330)
Supplement: Supplementary file 1 — Supporting Information [file ADVS-12-2501330-s001.docx]

**Supplementary Information**

**Turning Microstructure in Block copolymer Membranes: A Facile Strategy to Improve CO_2_ Separation Performance**

*Jing Wei, Min Deng, Zikang Qin, Weiyi Zhao, Yujie Li, Roman Selyanchyn, Hongyong Zhao, Jie Dong, Dengguo Yin, Yuanfa Zhuang,* *Liyuan Deng,* *Lin Yang, Lu Yao, Wenju Jiang, Junfeng Zheng, Bart Van der Bruggen, Zhongde Dai^*^*

Jing Wei, Min Deng

College of Architecture and Environment, Sichuan University, Chengdu 610065, China

National Engineering Research Centre for Flue Gas Desulfurization, Chengdu 610065, China

Carbon Neutral Technology Innovation Center of Sichuan, Chengdu 610065, China

College of Carbon Neutrality Future Technology, Sichuan University, Chengdu 610065, China

Zikang Qin

National Engineering Research Centre for Flue Gas Desulfurization, Chengdu 610065, China

Carbon Neutral Technology Innovation Center of Sichuan, Chengdu 610065, China

College of Carbon Neutrality Future Technology, Sichuan University, Chengdu 610065, China

Weiyi Zhao

National Synchrotron Radiation Laboratory University of Science and Technology of China, Hefei, 230029, P.R. China

Yujie Li

Imperial College London, Exhibition Rd, South Kensington, London SW7, UK

Roman Selyanchyn

Platform for Inter-/Transdisciplinary Energy Research (Q-PIT), Kyushu University, 744, Motooka, Nishi-ku, Fukuoka 819-0395, Japan

Hongyong Zhao, Jie Dong

State Key Laboratory of Separation Membranes and Membrane Processes/National Center for International Joint Research on Separation Membranes, Tiangong University, Tianjin 300387, China

School of Chemical Engineering and Technology, Tiangong University, Tianjin 300387, China

Dengguo Yin

College of Architecture and Environment, Sichuan University, Chengdu 610065, China

National Engineering Research Centre for Flue Gas Desulfurization, Chengdu 610065, China

Carbon Neutral Technology Innovation Center of Sichuan, Chengdu 610065, China

College of Carbon Neutrality Future Technology, Sichuan University, Chengdu 610065, China

DongFang Boiler Co.,Ltd., Zigong,643001, China

Yuanfa Zhuang

DongFang Boiler Co.,Ltd., Zigong,643001, China

Clean Combustion and Flue Gas Purification Key Laboratory of Sichuan Province, Deyang 618000, China

Liyuan Deng

Department of Chemical Engineering, Norwegian University of Science and Technology, Trondheim, 7491, Norway

Lin Yang, Lu Yao, Wenju Jiang, Junfeng Zheng,

National Engineering Research Centre for Flue Gas Desulfurization, Chengdu 610065, China

Carbon Neutral Technology Innovation Center of Sichuan, Chengdu 610065, China

College of Carbon Neutrality Future Technology, Sichuan University, Chengdu 610065, China

Bart Van der Bruggen

Department of Chemical Engineering, KU Leuven, Celestijnenlaan 200F, 3001, Leuven, Belgium

Zhongde Dai^*^

National Engineering Research Centre for Flue Gas Desulfurization, Chengdu 610065, China

Carbon Neutral Technology Innovation Center of Sichuan, Chengdu 610065, China

College of Carbon Neutrality Future Technology, Sichuan University, Chengdu 610065, China

*Corresponding Author:

Zhongde Dai: [zhongde.dai@scu.edu.cn](mailto:zhongde.dai@scu.edu.cn)

**Content**

[Materials 4](#_Toc191760684)

[Membrane characterization 5](#_Toc191760685)

[Figure S1. Chemical structure of Pebax^TM^ 2533 polymer (a) and optical photos of Pebax^TM^ 2533 membranes before and after MSR in GlyK solution (b). 8](#_Toc191760686)

[Figure S2. Schematic diagram of single gas permeation setup based on time-lag method. ^[2]^ 9](#_Toc191760687)

[Figure S3. Scheme of dry-state mixed-gas permeation test rig. ^[2]^ 10](#_Toc191760688)

[Figure S4. Flowchart of MSR HF Pebax^TM^/PVC TFC membrane module fabrication (a) and MSR flat-sheet Pebax^TM^/PAN TFC membrane fabrication (b). 11](#_Toc191760689)

[Table S1. The free volume parameters and lifetime spectrum information of the membrane before and after MSR. 12](#_Toc191760690)

[Table S2. FTIR peak assignments of Pebax^TM^ 2533 and GlyK 13](#_Toc191760691)

[Table S3. The activation energies of permeation for CO_2_ and N_2_ in MSR membranes compared to the original Pebax^TM^ 2533 14](#_Toc191760692)

[Figure S5. Effect of membrane thickness on Pebax^TM^ 2533-GlyK 10 wt.% membranes gas separation performance (Single gas, 2 bar, and 25 ^o^C). 15](#_Toc191760693)

[Figure S6. SAXS results of Pebax^TM^ 2533 membranes treated in ArgK solutions with different concentrations. 16](#_Toc191760694)

[Figure S7. XRD results of Pebax^TM^ 2533 membranes treated in ArgK solutions with different concentrations. 17](#_Toc191760695)

[Figure S8. TGA results of Pebax^TM^ 2533 membranes treated in ArgK solutions with different concentrations. 18](#_Toc191760696)

[Figure S9. DSC results of Pebax^TM^ 2533 membranes treated in ArgK solutions with different concentrations. 19](#_Toc191760697)

[Figure S10. FTIR spectra of Pebax^TM^ 2533 membranes treated in ArgK solutions with different concentrations. 20](#_Toc191760698)

[Figure S11. Salt uptake of Pebax^TM^ 2533 membranes treated in ArgK solutions with different concentrations. 21](#_Toc191760699)

[Figure S12. Gas separation performance of Pebax^TM^ 2533-ArgK membranes with different concentrations of ArgK. (Single gas, 2 bar, and 25 ^o^C). 22](#_Toc191760700)

[Figure S13. Effect of testing temperature on Pebax^TM^ 2533-ArgK membrane CO_2_ permeability and selectivity. 23](#_Toc191760701)

[Figure S14. Effect of feed pressure on Pebax^TM^ 2533-ArgK membrane CO_2_ permeability (a) and CO_2_/N_2_ selectivity (b) 24](#_Toc191760702)

[Figure S15. Gas separation performance of Pebax^TM^ 2533-ProK membranes with different concentrations of ProK. (Single gas, 2 bar, and 25 ^o^C). 25](#_Toc191760703)

[Figure S16. Effect of feed temperature on Pebax^TM^ 2533-ProK membrane CO_2_ permeability and selectivity. 26](#_Toc191760704)

[Figure S17. Effect of feed pressure on Pebax^TM^ 2533-ProK membrane CO_2_ permeability (a) and CO_2_/N_2_ selectivity (b). 27](#_Toc191760705)

[Figure S18. Chemical structure of Gly (a), Pro (b) and Arg (c). 28](#_Toc191760706)

[Figure S19. Long-term stability testing of Pebax^TM^ 2533-GlyK 10 wt.% membrane under a humid environment. (mixed gas, 2 bar, 25 ^o^C, and 100% RH). 29](#_Toc191760707)

[Table S4. CO_2_/N_2_ separation performance of different Pebax^TM^-based gas separation membranes, gas permeation data obtained via single gas permeation tests under dry conditions 30](#_Toc191760708)

[Figure S20. Impact of casting solution concentration on CO_2_ gas separation performance of flat sheet Pebax^TM^/PAN TFC membrane (mixed gas, 25 °C, 2 bar). 32](#_Toc191760709)

[Figure S21. Comparison of CO_2_ gas separation performance of HF membrane modules before and after MSR (mixed gas, 25 °C, 2 bar). 33](#_Toc191760710)

[Figure S22. CO_2_/N_2_ separation performance of HF membrane modules before and after MSR (mixed gas, 25 °C, 2 bar). 34](#_Toc191760711)

[Figure S23. Long-term stability testing of flat sheet Pebax^TM^/PAN TFC membranes (mixed gas, 25 °C, 2 bar). 35](#_Toc191760712)

[Figure S24. SEM images of the HF TFC membranes surfaces with different casting solution concentrations. 36](#_Toc191760713)

[Reference 37](#_Toc191760714)

# Materials

Pebax^TM^ 2533 was purchased from Arkema Co. Ltd (France). The molecular weights of polyamide 12 (PA12) and polytetramethylene oxide (PTMO) were 530 g mol^-1^ and 2000 g mol^-1^, respectively. In addition, the theoretical molar ratio of Pebax^TM^ 2533 is 9 mol% of PA12 and 91 mol% of PTMO.^[1]^ Ethanol was obtained from Adamas Co. Ltd (Shanghai, China). Laboratory-grade Deionized (DI) water was obtained from an ultra-water system (TE-S20, Hetai Instrument Co., Shanghai). Glycine (Gly), arginine (Arg), and proline (Pro) were acquired from Fuchen Chemical Reagent Co., Ltd (Tianjin, China). Stoichiometric amounts of three different amino acid salts in DI water were used to obtain amino acid salt solutions (GlyK, ArgK, and ProK) of the necessary concentrations. Polyvinyl chloride (PVC) hollow fiber (HF) porous support was purchased from Shenzhen Quanduoduo Technology Co., Ltd (Shenzhen, China). Polyacrylonitrile (PAN) porous support layer was provided by the Norwegian University of Science and Technology (NTNU, Norway). The CO_2_ and N_2_ gases (purity of 99.999%) as well as the 10:90 CO_2_/N_2_ mixed gas were all purchased from Chengdu Xuyuan Chemical Co., Ltd. (Chengdu, China).

# Membrane characterization

The microstructural changes of the membrane were characterized by transmission electron microscopy (TEM, Tecnai G2 F20, FEI, USA). The samples were exposed to a saturated lead acetate aqueous solution for selective staining, and subsequently embedded in epoxy resin and ultramicrotomed (Leica, UC6, Germany) at ambient temperature to yield electron-transparent sections.

The Positron Annihilation Lifetime Spectroscopy (PALS, DPLS3000, ORTEC, USA) was utilized to characterize the microvoids and pores within the membrane. PALS also provides quantitative information regarding free volume, thereby contributing to the understanding of material permeability and other physical properties. A ^22^Na positron source with an activity of approximately 2×10^6^ Bq was employed. During its β^+^ decay, positrons with kinetic energies of 0 to 540 keV are predominantly produced, almost simultaneously accompanied by the emission of 1.28 MeV gamma photons. The radioactive source was positioned between the samples, and a total of 2 million counts were measured to obtain the PALS for each sample. The system exhibits a time resolution of approximately 190 ps, with a channel width of 12.5 ps. The relation between the *o*-Ps lifetime and the radius of the free volume holes (*R*) followed the Equation S1:

$\tau_{3}=\frac{1}{2} [1-\frac{R}{R+\Delta R}+(\frac{1}{2\Pi})sin(\frac{2\Pi R}{R+\Delta R})]^{-1}$ (S1)

In the equation, *ΔR* represents the fitted empirical electron layer thickness, which is valued at 0.166 nm. Since the intensity, 𝐼_3_ indicates the number of free volume holes, the fractional free volume (*FFV*) can be calculated using the following empirical correlation:

$FFV=C\times V_{f}\times I_{3}=C\times\frac{4}{3}\Pi R^{3}\times I_{3}$ (S2)

The surface and cross-sectional morphologies of the prepared thin-film composite (TFC) membranes were observed using a field emission scanning electron microscope (SEM, Nova Nano SEM 450, FEI, USA). All cross-sectional samples were prepared by fracturing the samples in liquid nitrogen, and before imaging, all samples were gold-coated for 80 seconds.

The morphological details of samples were imaged using small angle X-ray scattering (SAXS, Nanostar U SAXS, Bruker, USA). The membranes were exposed to a 14 keV beam with a wavelength (λ) of 0.154 nm, and the sample-to-detector distance and spot size were 2 m and 0.5 x 0.5 mm, respectively. Intensity profiles were generated as a function of the scattering vector (q), where q = (4π/λ) sinθ and θ is the scattering half-angle, by azimuthally integrating the two-dimensional scattering patterns that were acquired.

The nanostructure of the membranes was also examined using a wide-angle X-ray diffraction (XRD, Rigaku Ultima IV, Rigaku, Japan) using Cu Kα radiation (λ = 1.5418 Å). The scanning range was set from 5 to 75°, with a scanning rate of 10 ^o^ min^-1^ and a step size of 0.02°.

The static contact angle (CA) was determined using the optical contact angle goniometer (DSA 100, Krüss GmbH, Germany). The sample was affixed to a glass slide to prevent curling, which could distort the test results. The slide was subsequently positioned on the sample stage, and ultrapure water (0.5 μL) was applied to the surface. The CA was automatically measured using the software.

The CO_2_ adsorption properties of the membrane, before and after MSR, were measured using the static volumetric method (H-Sorb 2600, Beishide, China). The CO_2_ adsorption and desorption behaviors were evaluated by varying the pressure in the range of 0–10 bar.

The elemental composition and chemical states of the sample surface were analyzed using X-ray photoelectron spectroscopy (XPS, Escalab Xi^+^, Thermo Fisher Scientific, USA). The instrument is equipped with a monochromatic Al Kα X-ray source (1486.6 eV).

The temperature-mass relationship of the samples from 30 to 800 °C was investigated using a simultaneous thermal analyzer (STA 449, Netzsch, Germany), with a heating rate of 10 ^o^C min^-1^. All measurements were performed under high-purity N_2_ (99.999%) at a flow rate of 60 mL min^-1^.

The thermal performance changes of the membranes before and after microstructure rearrangement (MSR) were analyzed using a differential scanning calorimeter (DSC 200 F3, Netzsch, Germany), specifically evaluating the *T_g_*. The testing temperature range was set from -150 °C to 150 °C, employing liquid nitrogen for cooling. Experiments were conducted under an N_2_ atmosphere to ensure an inert gas environment, thereby preventing oxidation and other reactions. The detailed temperature program was as follows: initially, heating from RT to 150 °C to remove moisture and other impurities, then cooling to -150 °C, followed by reheating to 150 °C, and finally cooling back to RT. The heating and cooling rates for each stage were 10 °C min^-1^. The *T_g_* of the membrane was determined using data from the second heating cycle. Before testing, samples underwent appropriate preparation and pre-treatment to ensure uniformity and consistency.

Fourier transform infrared spectroscopy (FTIR, Frontier, PerkinElmer, USA) was utilized for transmittance analysis and qualitative determination of surface chemical bonds in the samples, covering wavenumbers ranging from 650 to 4000 cm^-1^.

Non-solvent uptake of the membranes was conducted by immersing the samples into different types of non-solvent solutions for 24 hours at ambient conditions. The non-solvent uptake was calculated according to Equation (S3):

$\Omega_{salt}=\frac{W_{\infty}-W_{D}}{W_{D}}\times100$ (S3)

*W_∞_* and *W_D_* denote the weights of salt-saturated and dry membranes, respectively. The experimental error was below 5%, as calculated from the average values of these two samples.

**
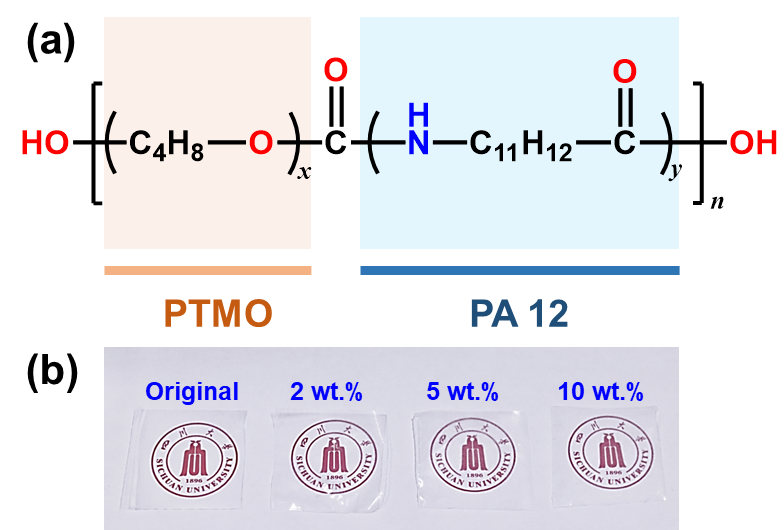
**

# **Figure S1**. Chemical structure of Pebax^TM^ 2533 polymer (a) and optical photos of Pebax^TM^ 2533 membranes before and after MSR in GlyK solution (b).


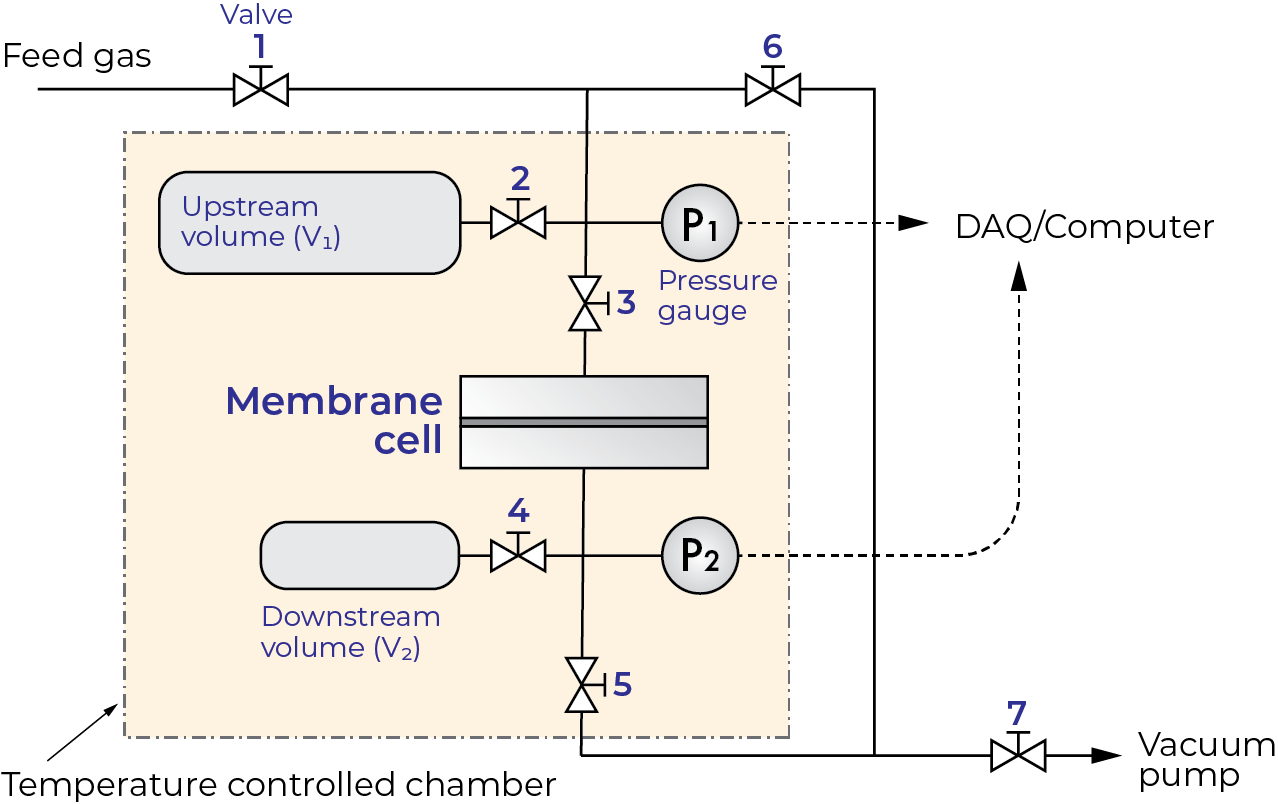


# **Figure S2**. Schematic diagram of single gas permeation setup based on time-lag method. ^[2]^

**Note S2:** The single gas permeability obtained by constant volume-variable pressure method on a low-pressure membrane permeation system (LP1) (Xuzhou North Gaorui Electronic Equipment Co., LTD.). Before the gas permeation test, the membrane was first sealed in the membrane pool. The pressure on both the feed and permeate sides of the membrane was vacuumed to less than 5 Pa using a vacuum pump. Subsequently, gas was introduced into the feed side, and gas permeation tests were initiated. The feed gas pressure varied between 2 to 6 bar, while the testing temperature ranged from 25 to 45 ^o^C. To ensure the accuracy and repeatability of the test results, each membrane was tested at least three times.


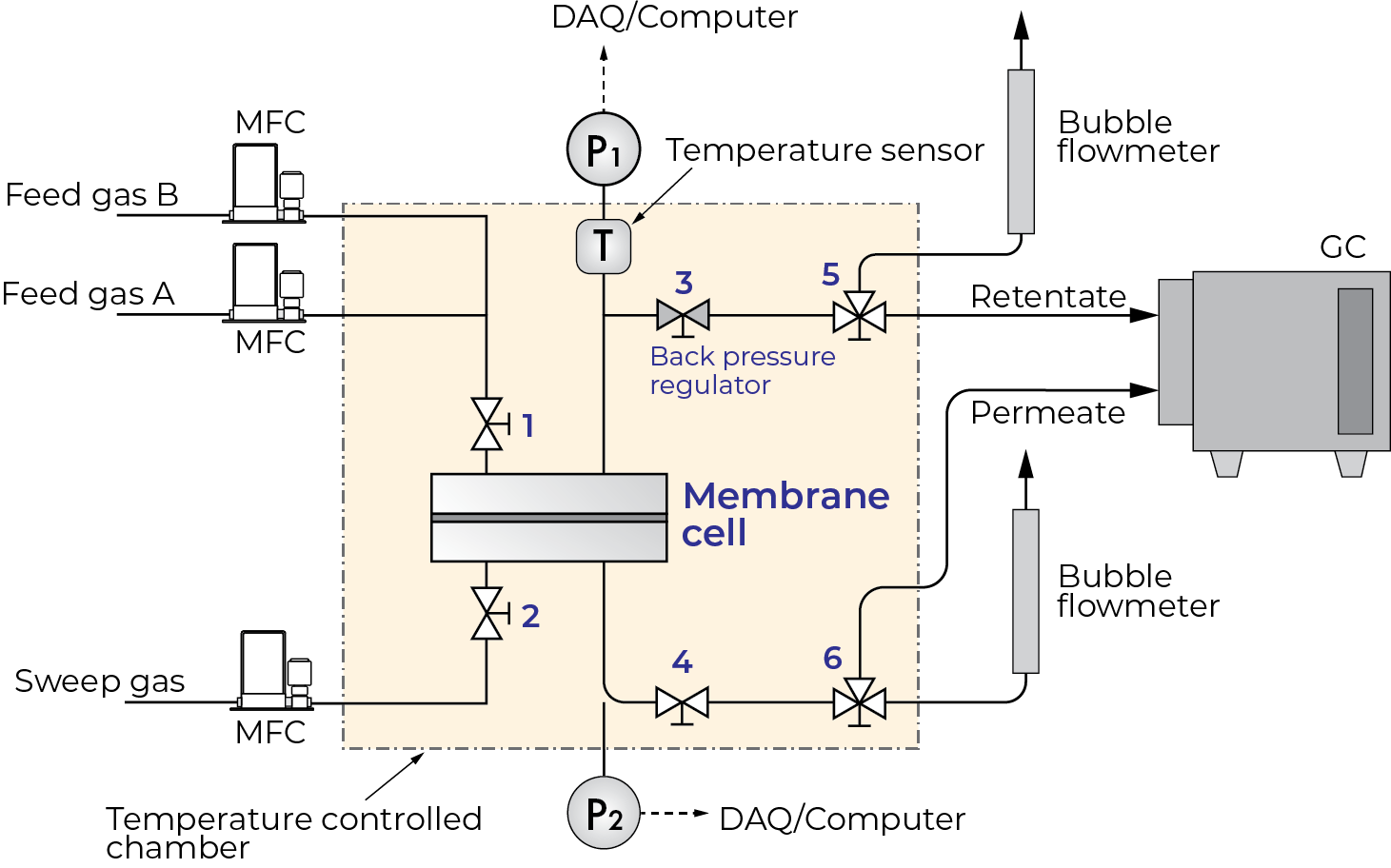


# Figure S3. Scheme of dry-state mixed-gas permeation test rig. ^[2]^

**Note S3:** The CO_2_/N_2_ binary gas mixture (10/90 vol%) was employed as the feed gas to investigate the CO_2_/N_2_ separation performance in a mixed-gas membrane separation system (Nanjing Hopu Analytical Instrument Co., Ltd.). Helium was used as the sweep gas, and the feed gas pressure (2–6 bar) and testing temperature (25–45 °C) were controlled according to the experimental conditions. The gas composition on the permeate side was analyzed and monitored using a gas chromatograph (GC 9790 Plus, Fuli, China).

**
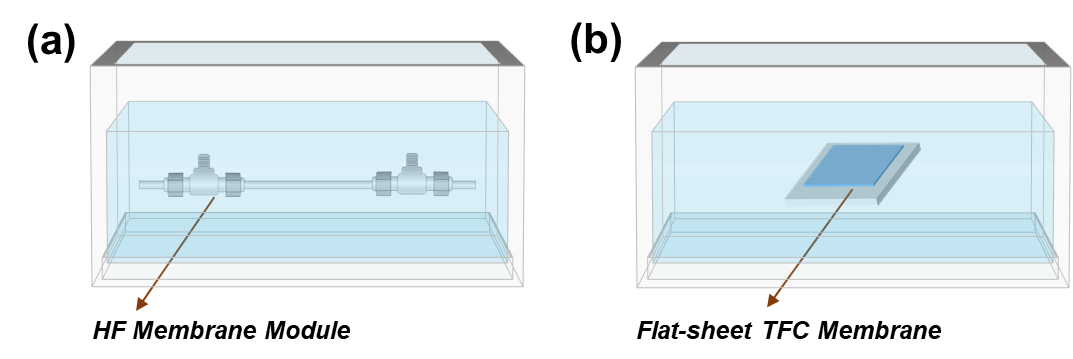
**

# Figure S4. Flowchart of MSR HF Pebax^TM^/PVC TFC membrane module fabrication (a) and MSR flat-sheet Pebax^TM^/PAN TFC membrane fabrication (b).

# Table S1. The free volume parameters and lifetime spectrum information of the membrane before and after MSR.

| **Free volume parameters between polymer chains of membranes** | | | | |
| --- | --- | --- | --- | --- |
| **Membranes** | $\tau_{3}$ (ns) | *I_3_* (%) | *R* (Å) | *FFV* (%) |
| Original Pebax^TM^ 2533 | 2.64 | 23.2 | 3.37 | 3.73 |
| Pebax^TM^ 2533-DI Water | 2.62 | 23.3 | 3.36 | 3.69 |
| **The information on the first and second-lifetime spectra** | | | | |
| **Membranes** | $\tau_{1}$ (ns) | *I_1_* (%) | $\tau_{2}$ (ns) | *I_2_* (%) |
| Original Pebax^TM^ 2533 | 0.184 | 24.8 | 0.416 | 52.0 |
| Pebax^TM^ 2533-DI Water | 0.200 | 29.0 | 0.423 | 47.4 |

# Table S2. FTIR peak assignments of Pebax^TM^ 2533 and GlyK

| **Wavenumber (cm^-1^)** | | **Peak assignment** |
| --- | --- | --- |
| Pebax 2533 | GlyK |  |
| - | 895 | stretching of CCN |
| 1106 | - | stretching of -C-O-C- |
|  | 1302 | twisting of -CH_2_ |
| 1538 | - | bending of N-H |
| - | 1564 | stretching of -COO |
| 1638 | - | stretching of -C=O in H-N-C=O |
| 1734 | - | stretching of -C=O |
| 2853 | - | bending of -C-H |
| 2923 | - | bending of -C-H |
| 3305 | 3431 | stretching of N-H, stretching of -OH |

# Table S3. The activation energies of permeation for CO_2_ and N_2_ in MSR membranes compared to the original Pebax^TM^ 2533

|  | **Ep (kJ/mol)** | |
| --- | --- | --- |
| **Membranes** | CO_2_ | N_2_ |
| Pebax^TM^ 2533 | 16.3 | 25.6 |
| Pebax^TM^ 2533- DI water | 10.1 | 24.5 |
| Pebax^TM^ 2533-GlyK 10 wt.% | 17.3 | 23.1 |
| Pebax^TM^ 2533-ProK 2 wt.% | 13.1 | 24.7 |
| Pebax^TM^ 2533-ArgK 5 wt.% | 14.5 | 29.3 |

**
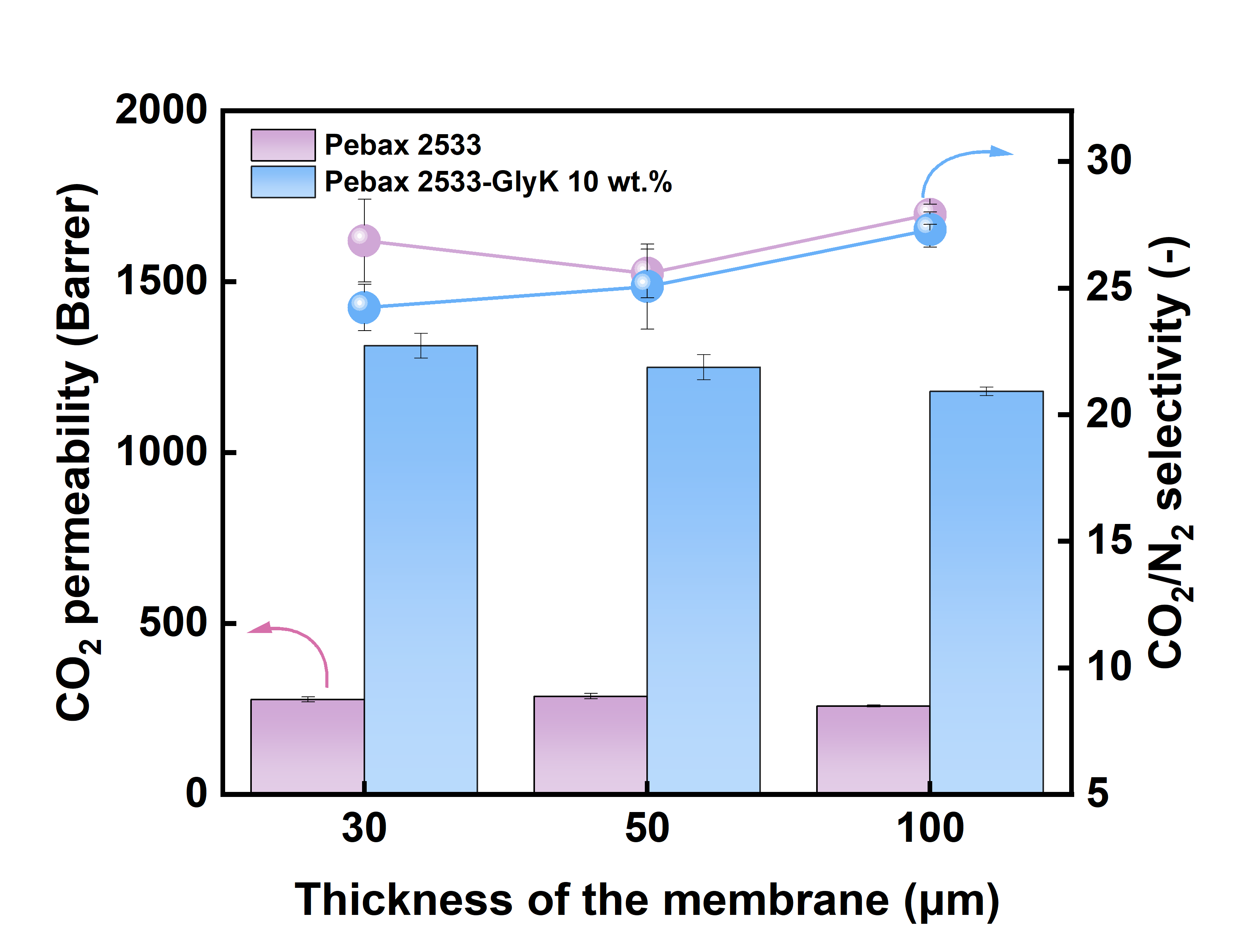
**

# Figure S5. Effect of membrane thickness on Pebax^TM^ 2533-GlyK 10 wt.% membranes gas separation performance (Single gas, 2 bar, and 25 ^o^C).

**Note S5.** The effect of the membrane thickness was also investigated to check the effectiveness of the MSR. As indicated in Figure. S5, the MSR works similarly for self-standing membranes with a thickness range of 30-100 μm. In addition, the MSR is more effective in promoting the CO_2_ permeability for the membranes with lower thicknesses (1313 Barrer for 30 μm and 1179 Barrer for 100 μm), clearly showing its potential in promoting CO_2_ permeance in TFC membranes.

**
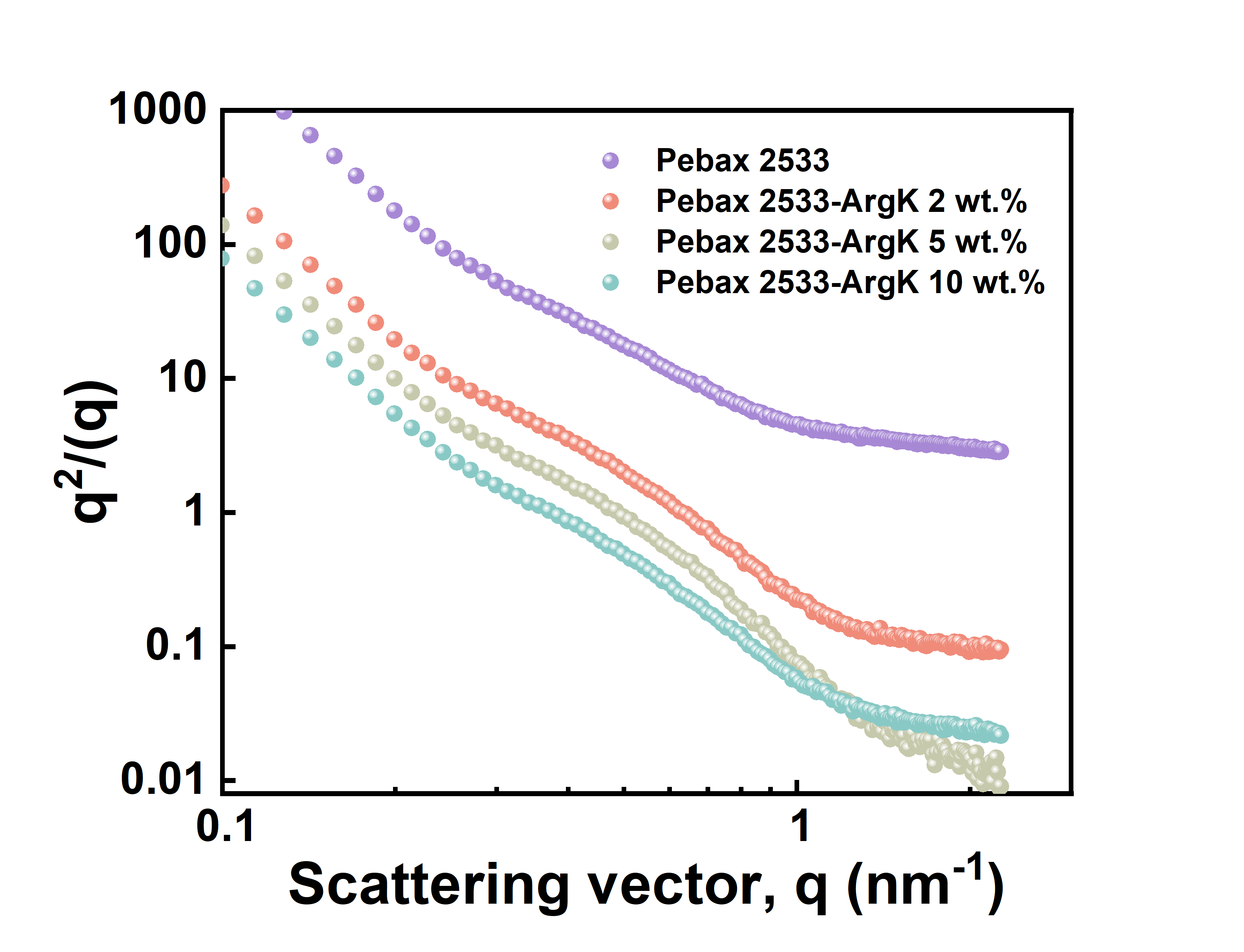
**

# Figure S6. SAXS results of Pebax^TM^ 2533 membranes treated in ArgK solutions with different concentrations.

**
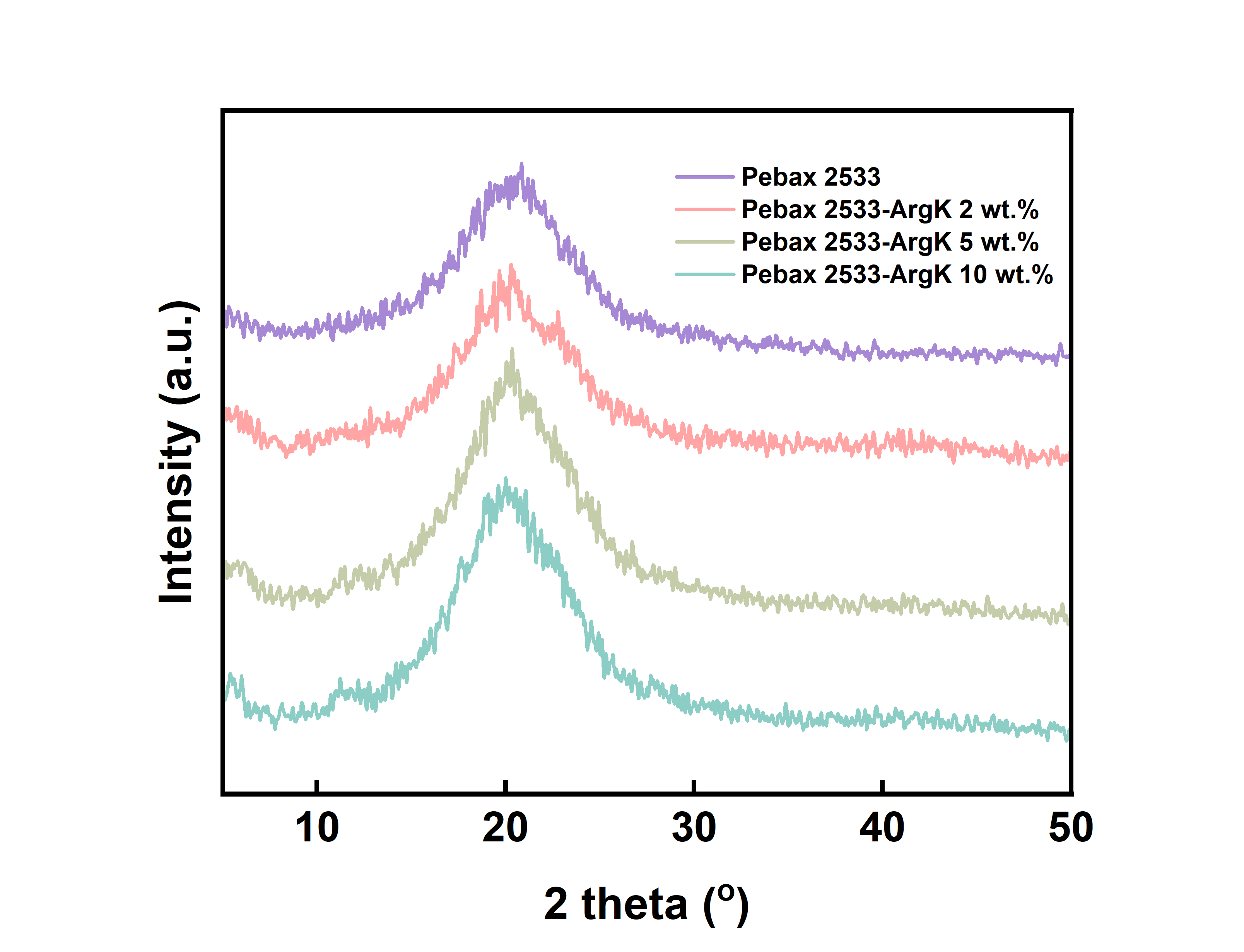
**

# Figure S7. XRD results of Pebax^TM^ 2533 membranes treated in ArgK solutions with different concentrations.

**
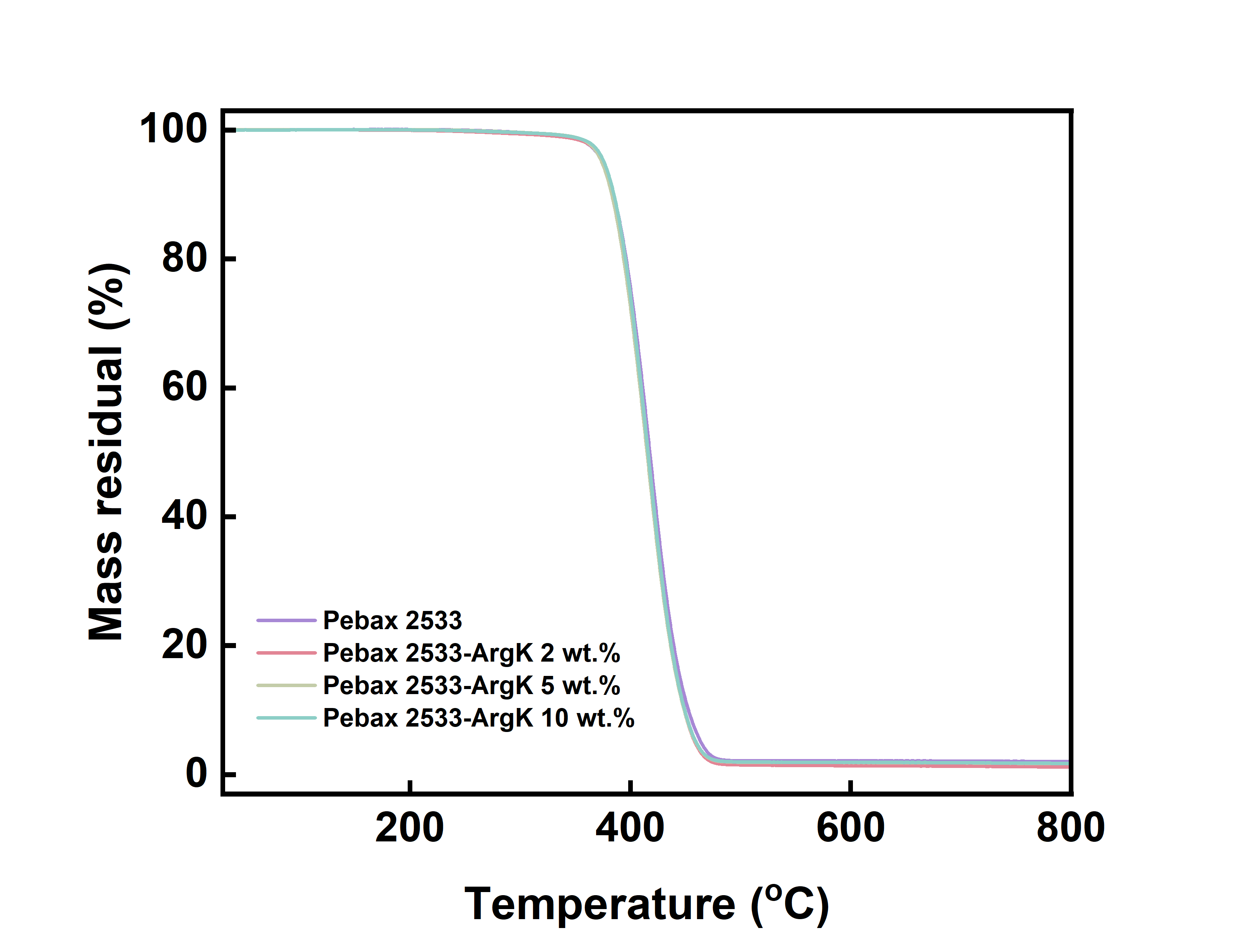
**

# Figure S8. TGA results of Pebax^TM^ 2533 membranes treated in ArgK solutions with different concentrations.

**
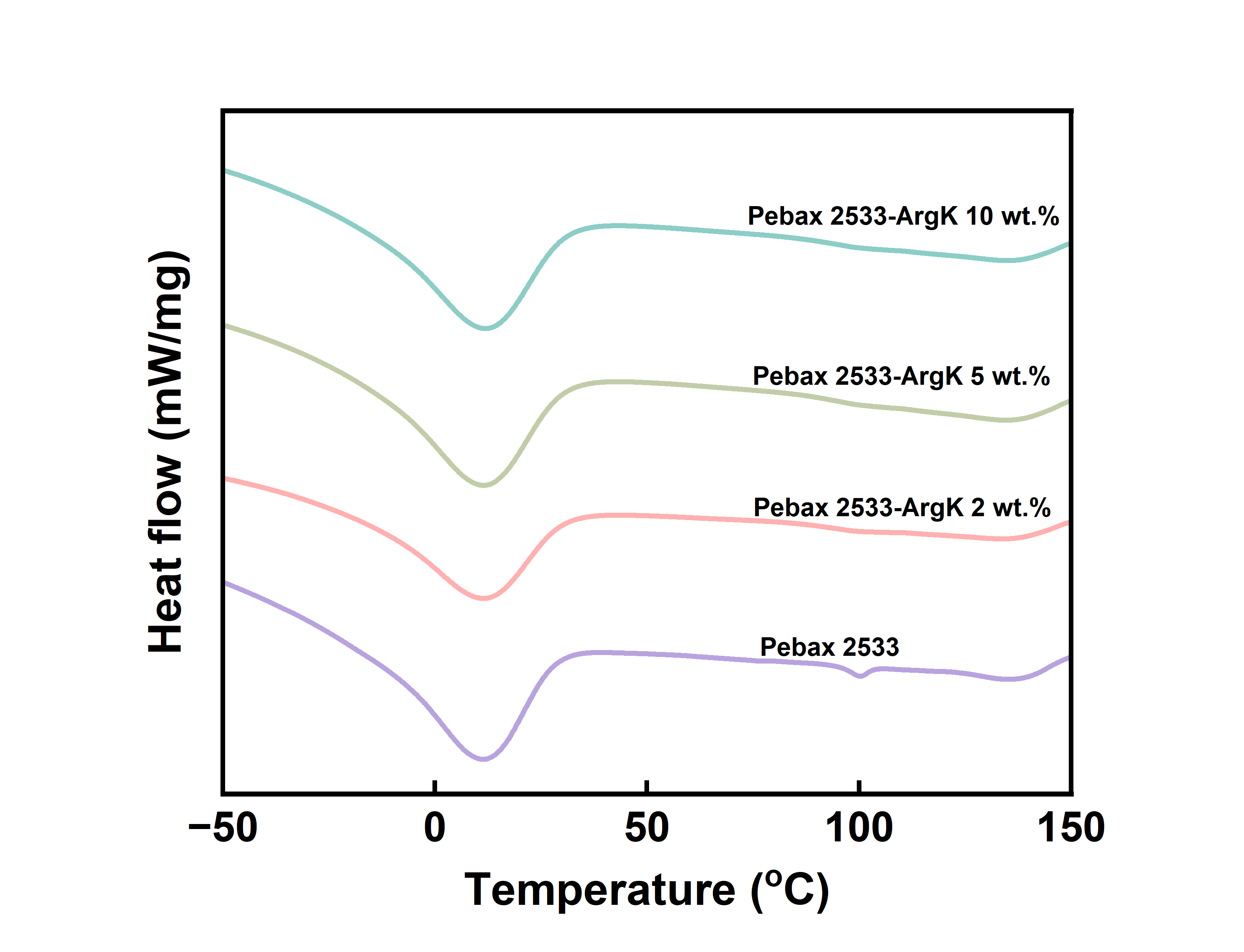
**

# Figure S9. DSC results of Pebax^TM^ 2533 membranes treated in ArgK solutions with different concentrations.

**
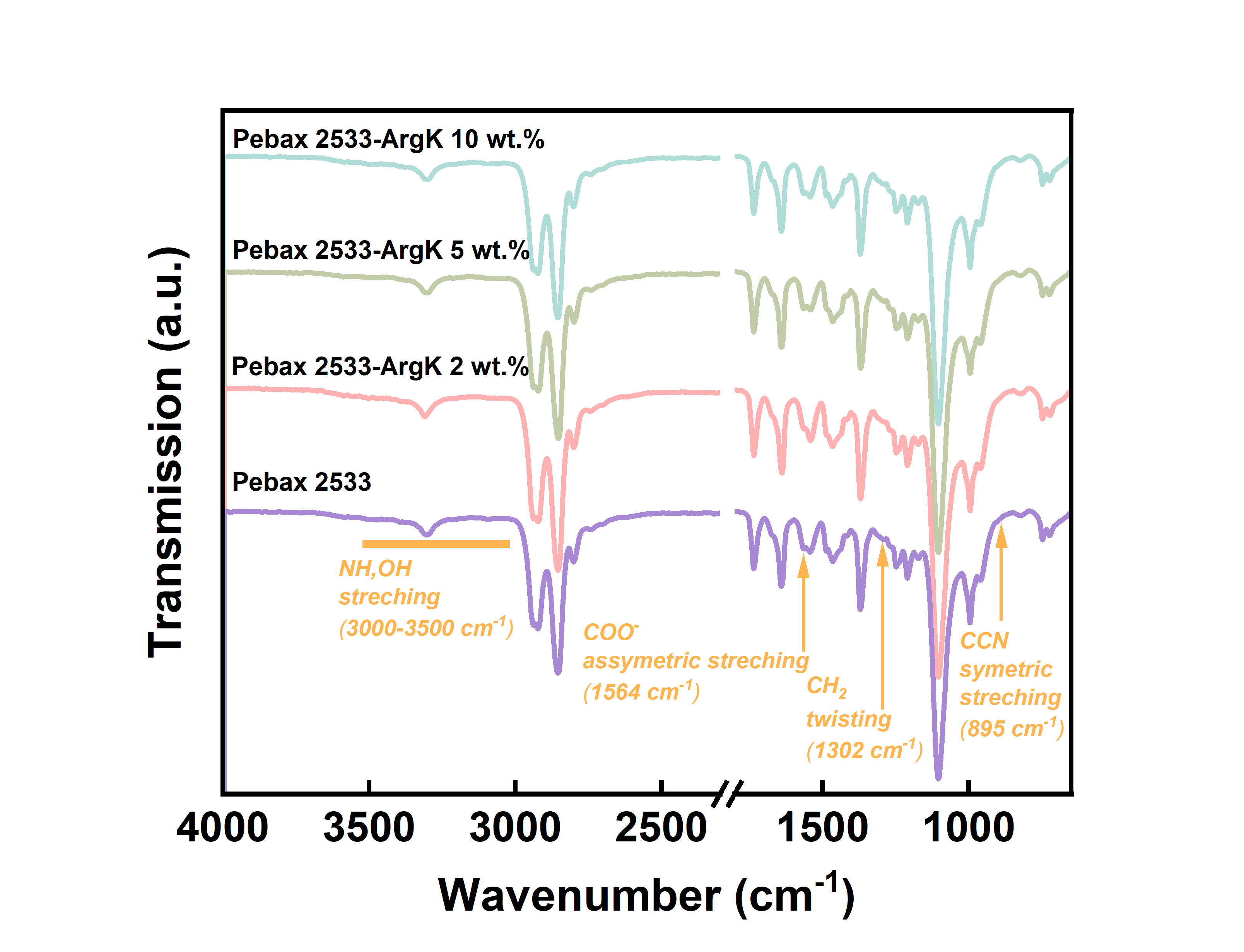
**

# Figure S10. FTIR spectra of Pebax^TM^ 2533 membranes treated in ArgK solutions with different concentrations.

**
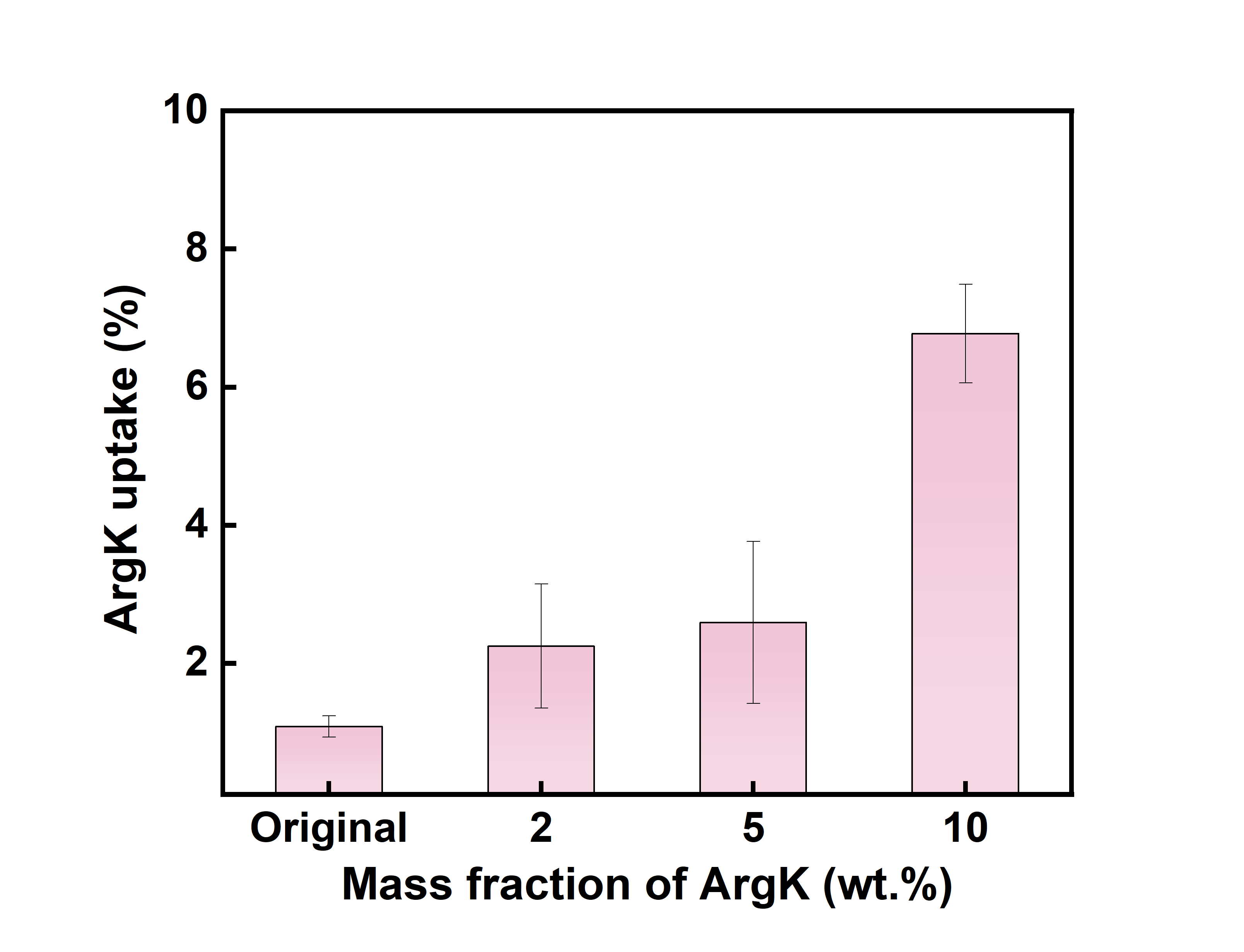
**

# Figure S11. Salt uptake of Pebax^TM^ 2533 membranes treated in ArgK solutions with different concentrations.


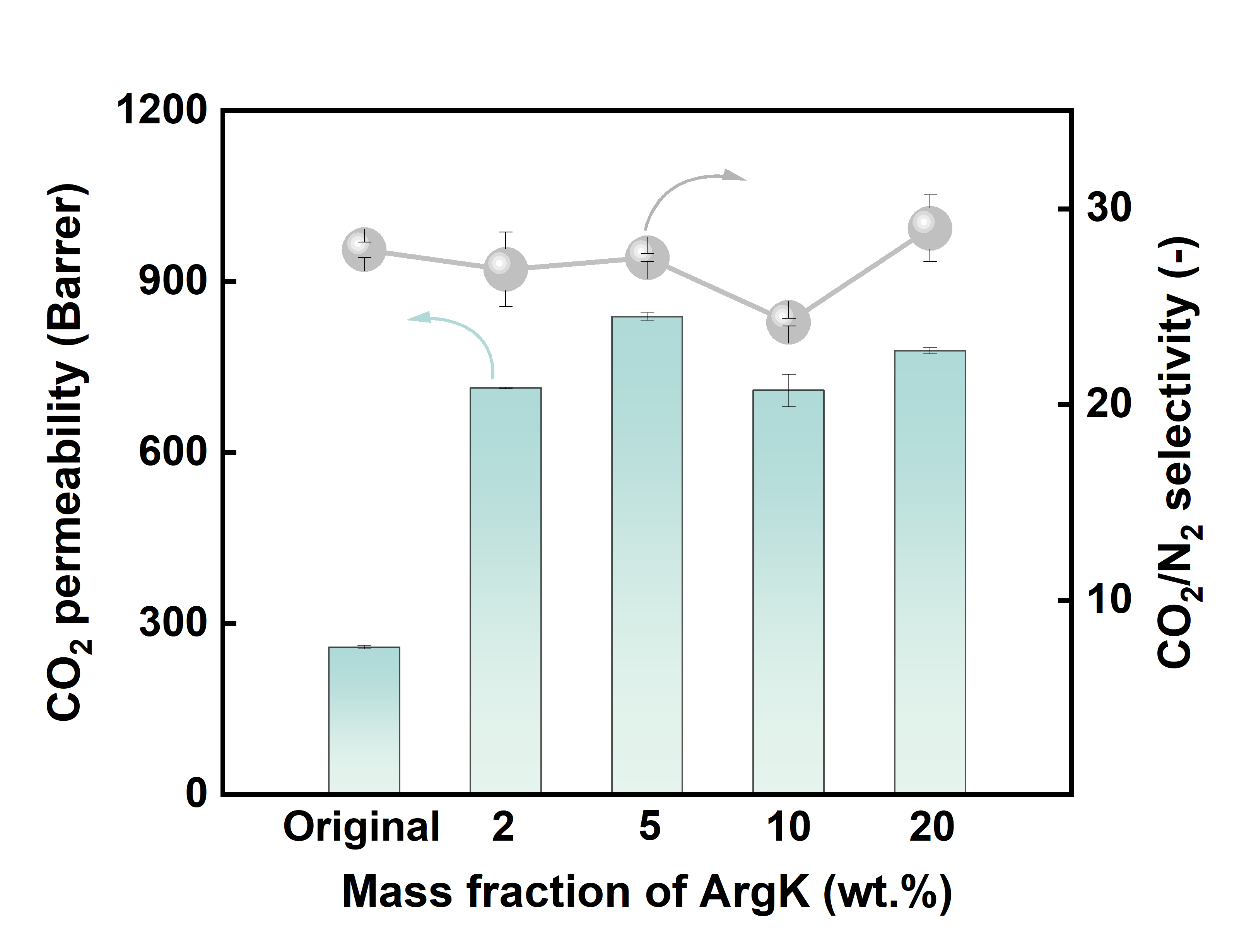


# **Figure S12**. Gas separation performance of Pebax^TM^ 2533-ArgK membranes with different concentrations of ArgK. (Single gas, 2 bar, and 25 ^o^C).

**Note S12:** We also immersed the Pebax^TM^ 2533 membrane in ArgK solution with different concentrations to explore the effects of ArgK concentrations. CO_2_/N_2_ separation results are shown in Figure S12. The results revealed that the maximum CO_2_ permeability reached 838.8 Barrer at a concentration of 5 wt.% ArgK. As the salt concentration increased, CO_2_ permeability gradually decreased, mirroring the effect observed with increasing ProK solution concentration.

**
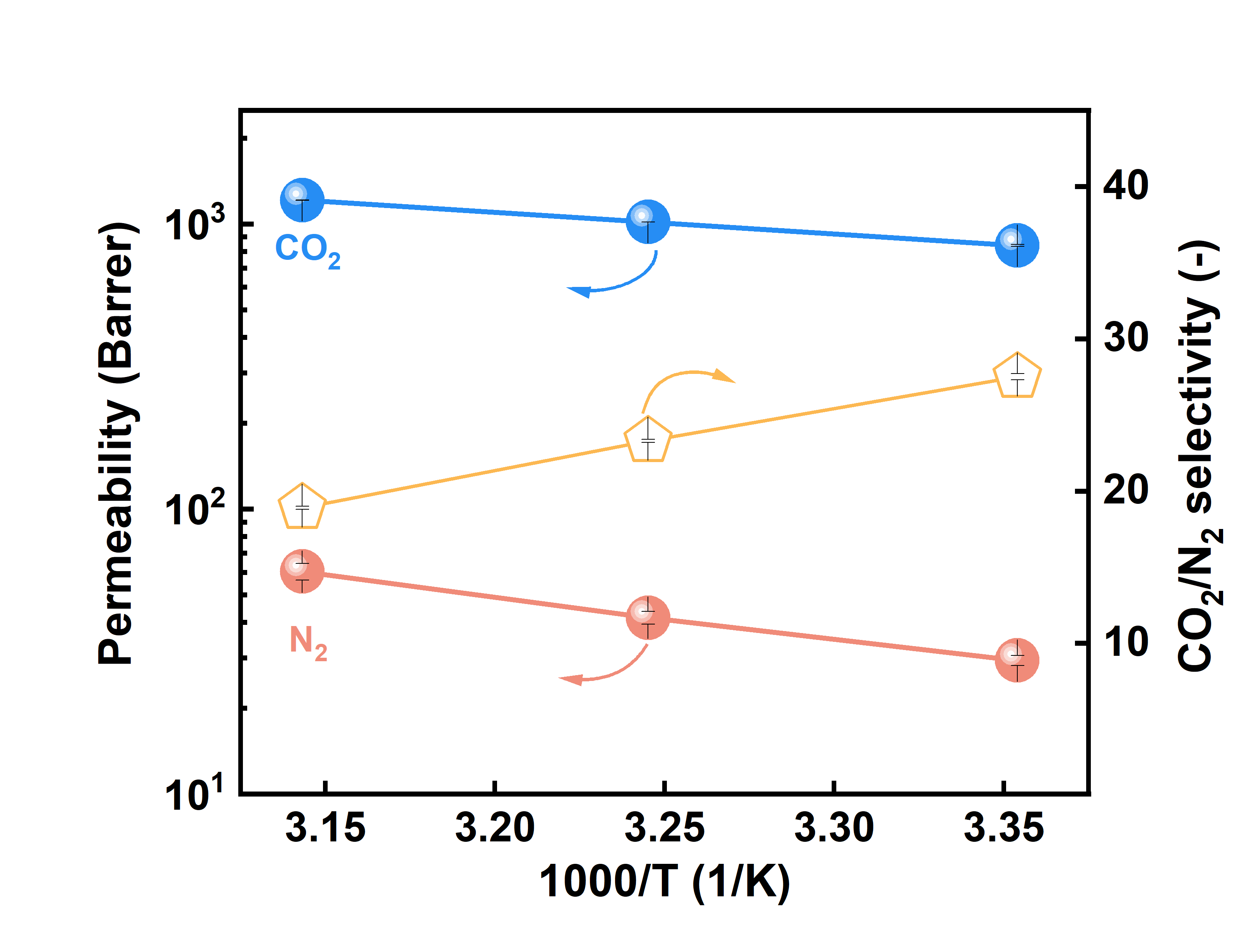
**

# Figure S13. Effect of testing temperature on Pebax^TM^ 2533-ArgK membrane CO_2_ permeability and selectivity.

**Note S13:** The influence of temperature was investigated in the range of 25-45 °C. It was observed that, similar to most polymeric membranes based on solution-diffusion model, as the operating temperature increased from 25 °C to 45 °C, the CO_2_ permeability of the membrane increased progressively from 838.8 Barrer to 1210.8 Barrer. However, the selectivity of CO_2_/N_2_ decreased significantly from 27.5 to 18.9.

**
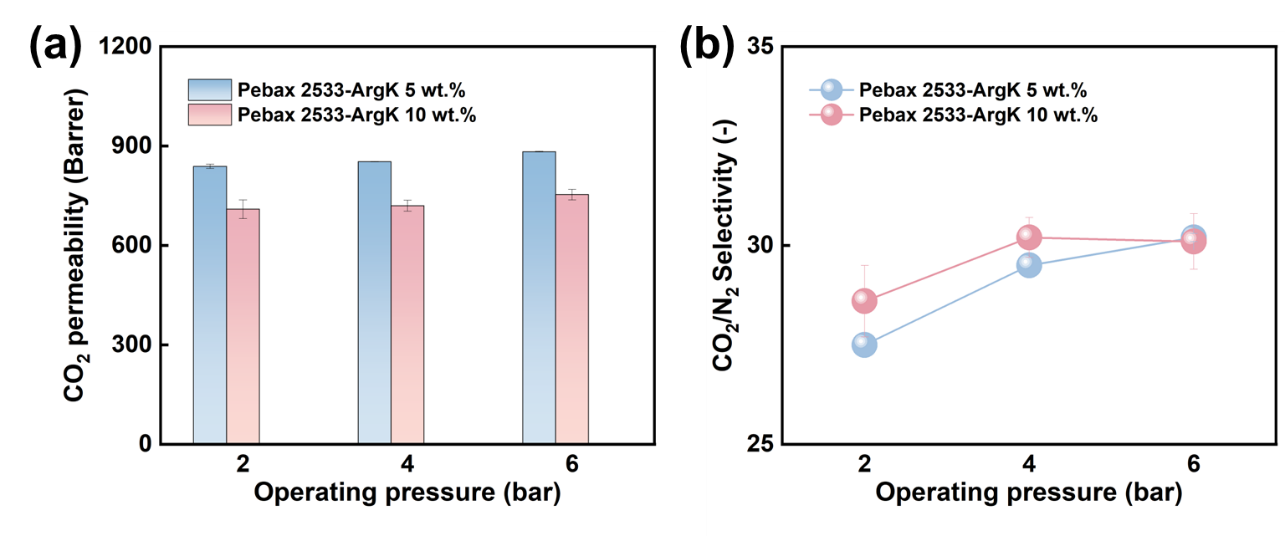
**

# Figure S14. Effect of feed pressure on Pebax^TM^ 2533-ArgK membrane CO_2_ permeability (a) and CO_2_/N_2_ selectivity (b)

**Note S14:** Furthermore, Figure S14 (a) and (b) illustrate the CO_2_ separation performance of Pebax^TM^ 2533-ArgK membranes at feed pressures of 2, 4, and 6 bar. As observed from the figures, as the pressure increases from 2 bar to 6 bar, the CO_2_ permeability of the membrane increases slowly, while the CO_2_/N_2_ selectivity remains stable.

**
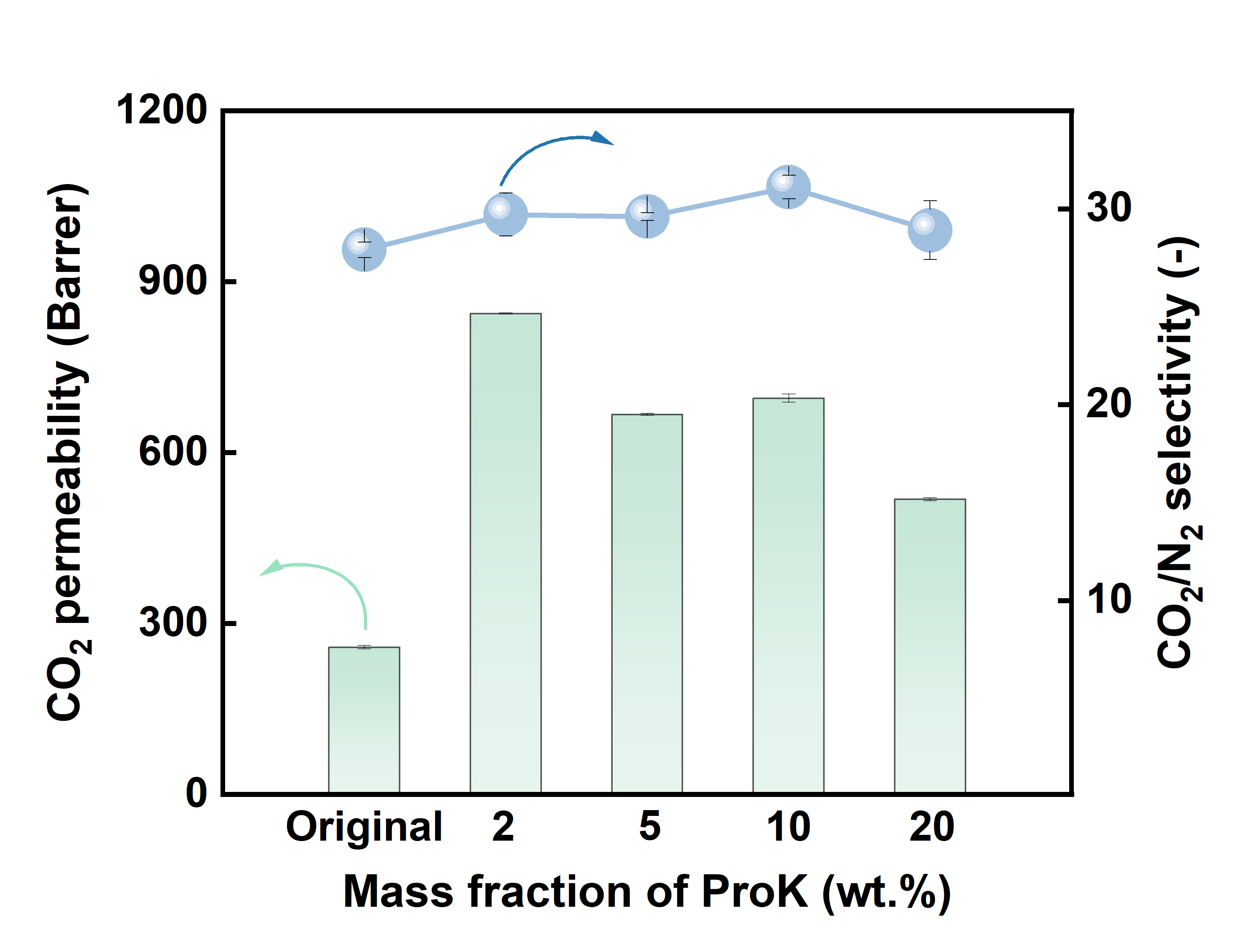
**

# Figure S15. Gas separation performance of Pebax^TM^ 2533-ProK membranes with different concentrations of ProK. (Single gas, 2 bar, and 25 ^o^C).

**Note S15:** The CO_2_ separation performance of Pebax^TM^ 2533-ProK membranes was investigated, and the corresponding results are presented in Figure S15. The data reveal that the maximum CO_2_ permeability (844.4 Barrer) was obtained with a ProK concentration of 2 wt.%. With the increase of ProK concentration, the permeability of CO_2_ gradually decreased, which may be due to the excessive salt crystals blocking the transport channels of the Pebax^TM^ 2533 membrane.

**
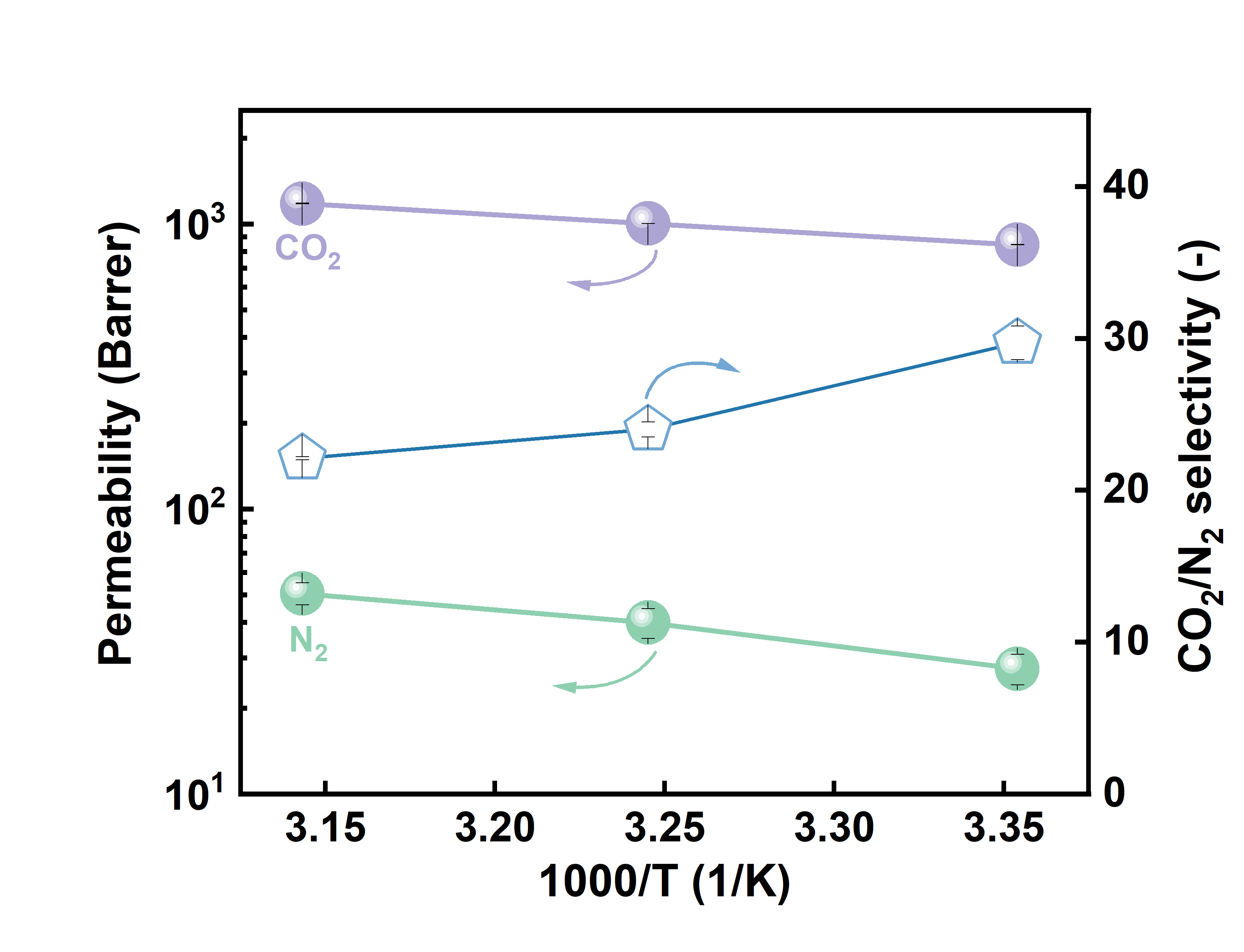
**

# Figure S16. Effect of feed temperature on Pebax^TM^ 2533-ProK membrane CO_2_ permeability and selectivity.

**Note S16:** Furthermore, the effect of test temperature on the CO_2_/N_2_ separation performance was investigated. The effect of temperature was examined within the range of 25 to 45 ^o^C. Like many CO_2_-selective membranes, ^[3-4]^ as the operation temperature increased from 25 ^o^C to 45 ^o^C, the CO_2_ permeability of the membrane gradually increased from 844.4 Barrer to 1178.1 Barrer. On the other hand, increasing the test temperature results in a decrease in CO_2_/N_2_ selectivity. In this study, as the test temperature increased from 25 °C to 45 °C, the CO_2_/N_2_ selectivity was reduced from 29.7 to 22.1.

**
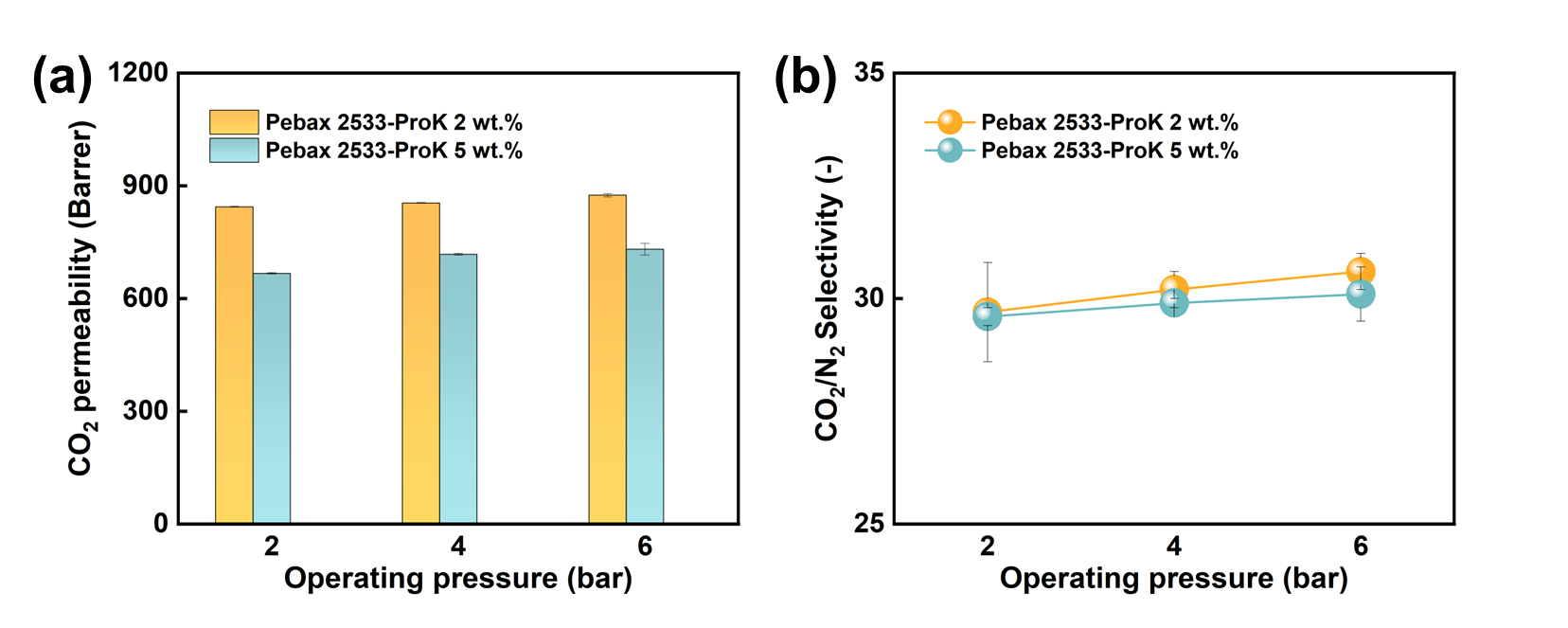
**

# Figure S17. Effect of feed pressure on Pebax^TM^ 2533-ProK membrane CO_2_ permeability (a) and CO_2_/N_2_ selectivity (b).

**Note S17:** Furthermore, Figure. S17 (a) and S17 (b) show the CO_2_ separation performance of Pebax^TM^ 2533-ProK 2 wt.% membrane with feed pressures of 2, 4, and 6 bar. As can be seen from the figure, with the pressure increases, the CO_2_ permeability showed a slow rising trend, which was consistent with the literature reports.^[5-7]^ The CO_2_/N_2_ selectivity remained stable, indicating that the ProK salt solution-treated Pebax^TM^ 2533 and original Pebax^TM^ 2533 membrane have similar CO_2_ separation behavior.


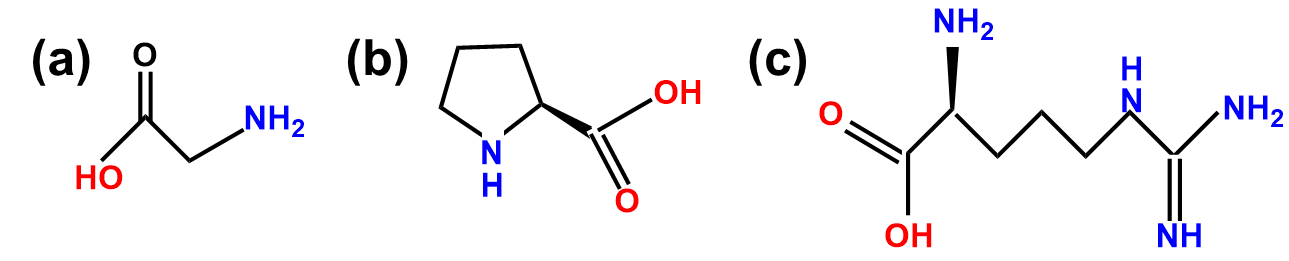


# Figure S18. Chemical structure of Gly (a), Pro (b) and Arg (c).


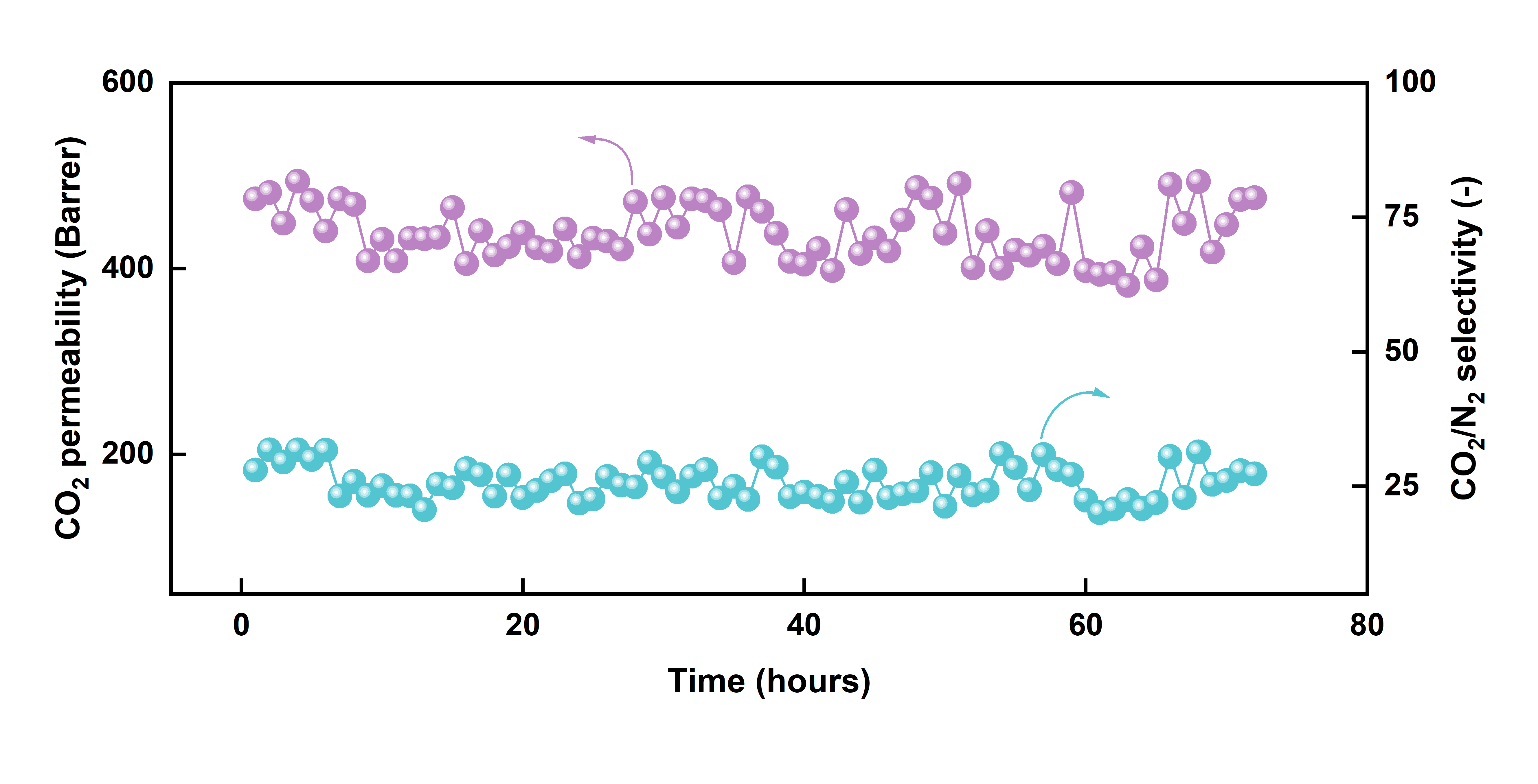


# Figure S19. Long-term stability testing of Pebax^TM^ 2533-GlyK 10 wt.% membrane under a humid environment. (mixed gas, 2 bar, 25 ^o^C, and 100% RH).

**Note S19:** The Pebax^TM^ 2533-GlyK 10 wt.% self-standing membrane was evaluated under humidified mixed gas conditions (CO_2_/N_2_ 10:90, 100% RH) for 72 hours. Surprisingly, Different from many rubbery polymers (Nafion ^[8]^, Polyactive ^[9]^ and PEG ^[10]^), normally the presence of the water vapor will improve the CO_2_ permeability, surprisingly, in the current study, the MSR membrane exhibited a behaver more like highly permeable glassy polymers (e.g., PIM ^[11]^, PTMSP ^[12]^), the CO_2_ permeabiltiy was lower under humid conditions compared to the dry state results. a $P_{{CO}_{2}}$ of 469.7 Barrer and $\alpha_{{CO}_{2}/N_{2}}$ of 28.9 was documented. In addition, Pebax^TM^ 2533-GlyK 10 wt.% membrane showed excellent stability in terms of CO_2_ permeability and CO_2_/N_2_ selectivity throughout the test period. The reason behind these phenomena is still unclear, more in-depth analysis and powerful tool like synchrotron radiation will be employed to characterize the membrane in future work.

# Table S4. CO_2_/N_2_ separation performance of different Pebax^TM^-based gas separation membranes, gas permeation data obtained via single gas permeation tests under dry conditions

| **Membrane** | **Test conditions** | **P_CO2_ (Barrer)** | $\text{α}_{\text{CO}_{\text{2}}\text{/}\text{N}_{\text{2}}}\boldsymbol{(-)}$ | **Ref.** |
| --- | --- | --- | --- | --- |
| Pebax^TM^ 2533-(23 wt.%ZIF-7-OH) | 4.5 bar / 25 °C | 323 | 13.7 | ^[13]^ |
| Pebax^TM^ 2533-(14 wt.%ZIF-7-OH) | 4.5 bar / 25 °C | 272.3 | 37.8 | ^[13]^ |
| Pebax^TM^ 2533-(8 wt.%ZIF-7-OH) | 4.5 bar / 25 °C | 247.0 | 32.4 | ^[13]^ |
| Pebax^TM^ 2533-(2 wt.%ZIF-7-OH) | 4.5 bar / 25 °C | 249 | 32.5 | ^[13]^ |
| Pebax^TM^ 2533- (70 wt.% ZIF-11) | 2 bar / 20 °C | 402. | 28.0 | ^[14]^ |
| Pebax^TM^ 2533-(30 wt.% PEG-POSS) | 10 bar / 30 °C | 298.4 | 29.8 | ^[15]^ |
| Pebax^TM^ 2533-(40 wt.% PEG-POSS) | 10 bar / 30 °C | 288.3 | 30.0 | ^[15]^ |
| Pebax^TM^ 2533-(50 wt.% PEG-POSS) | 10 bar / 30 °C | 146.3 | 34.8 | ^[15]^ |
| Pebax^TM^ 2533- (5 wt.% ZIF-8) | 2 bar / 25 °C | 385.0 | 29.6 | ^[16]^ |
| Pebax^TM^ 2533- (10 wt.% ZIF-8) | 6 bar / 25 °C | 433.0 | 30.9 | ^[16]^ |
| Pebax^TM^ 2533- (20 wt.% TCP) | 1 bar / 35 °C | 513.3 | 21.8 | ^[17]^ |
| Pebax^TM^ 2533- (0.02 wt.% PGO) | 1 bar / 35 °C | 397.4 | 23.8 | ^[18]^ |
| Pebax^TM^ 2533- (0.02 wt.% PEAGO) | 1 bar / 35 °C | 380.4 | 24.2 | ^[18]^ |
| Pebax^TM^ 2533- (10 wt.% 0D ZIF) | 6 bar / 25 °C | 375.5 | 31.4 | ^[19]^ |
| Pebax^TM^ 2533- (10 wt.% 1D ZIF) | 6 bar / 25 °C | 398.8 | 17.6 | ^[19]^ |
| Pebax^TM^ 2533- (5 wt.% 2D ZIF) | 6 bar / 25 °C | 410.4 | 26.9 | ^[19]^ |
| Pebax^TM^ 2533- (5 wt.% [TMGH][Im]) | 1 bar / 23 °C | 139.0 | 11.0 | ^[20]^ |
| Pebax^TM^ 2533- (10 wt.%[TMGH][Im]) | 1 bar / 23 °C | 200.1 | 14.5 | ^[20]^ |
| Pebax^TM^ 2533- (15 wt.% [TMGH][Im]) | 1 bar / 23 °C | 214.0 | 14.0 | ^[20]^ |
| Pebax^TM^ 2533- (20 wt.% [Bmim][CF_3_SO_3_] | 1 bar / 25 °C | 272.2 | 33.3 | ^[21]^ |
| Pebax^TM^ 2533- (40 wt.% [Bmim][CF_3_SO_3_] | 1 bar / 25 °C | 307.7 | 30.8 | ^[21]^ |
| Pebax^TM^ 2533- (60 wt.% [Bmim][CF_3_SO_3_] | 1 bar / 25 °C | 297.3 | 31.0 | ^[21]^ |
| Pebax^TM^ 2533 | 2 bar / 25 °C | 258.5 | 27.9 | This work |
| Pebax^TM^ 2533- DI Water | 2 bar / 25 °C | 782.3 | 30.8 | This work |
| Pebax^TM^ 2533-GlyK 10 wt.% | 2 bar / 25 °C | 1179.5 | 27.3 | This work |
| Pebax^TM^ 2533-ProK 2 wt.% | 2 bar / 25 °C | 844.4 | 29.7 | This work |
| Pebax^TM^ 2533-ArgK 5 wt.% | 2 bar / 25 °C | 838.8 | 27.5 | This work |
| Nafion D520 | 2 bar / 25 °C/100% RH | 209.3 | 13.6 | This work |
| Nafion D520-24 h-25 ^o^C | 2 bar / 25 °C//100% RH | 472.0 | 60.6 | This work |


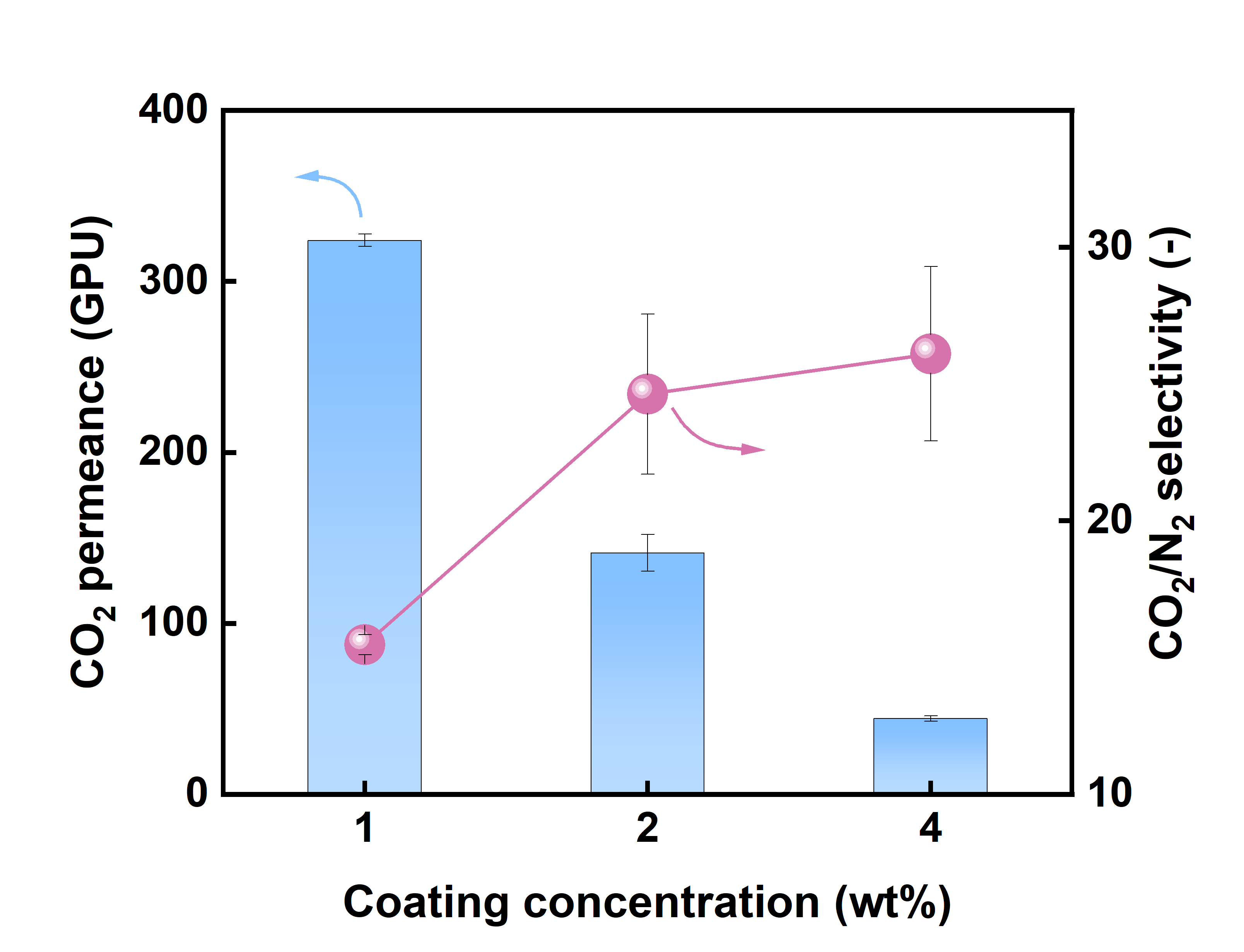


# Figure S20. Impact of casting solution concentration on CO_2_ gas separation performance of flat sheet Pebax^TM^/PAN TFC membrane (mixed gas, 25 °C, 2 bar).


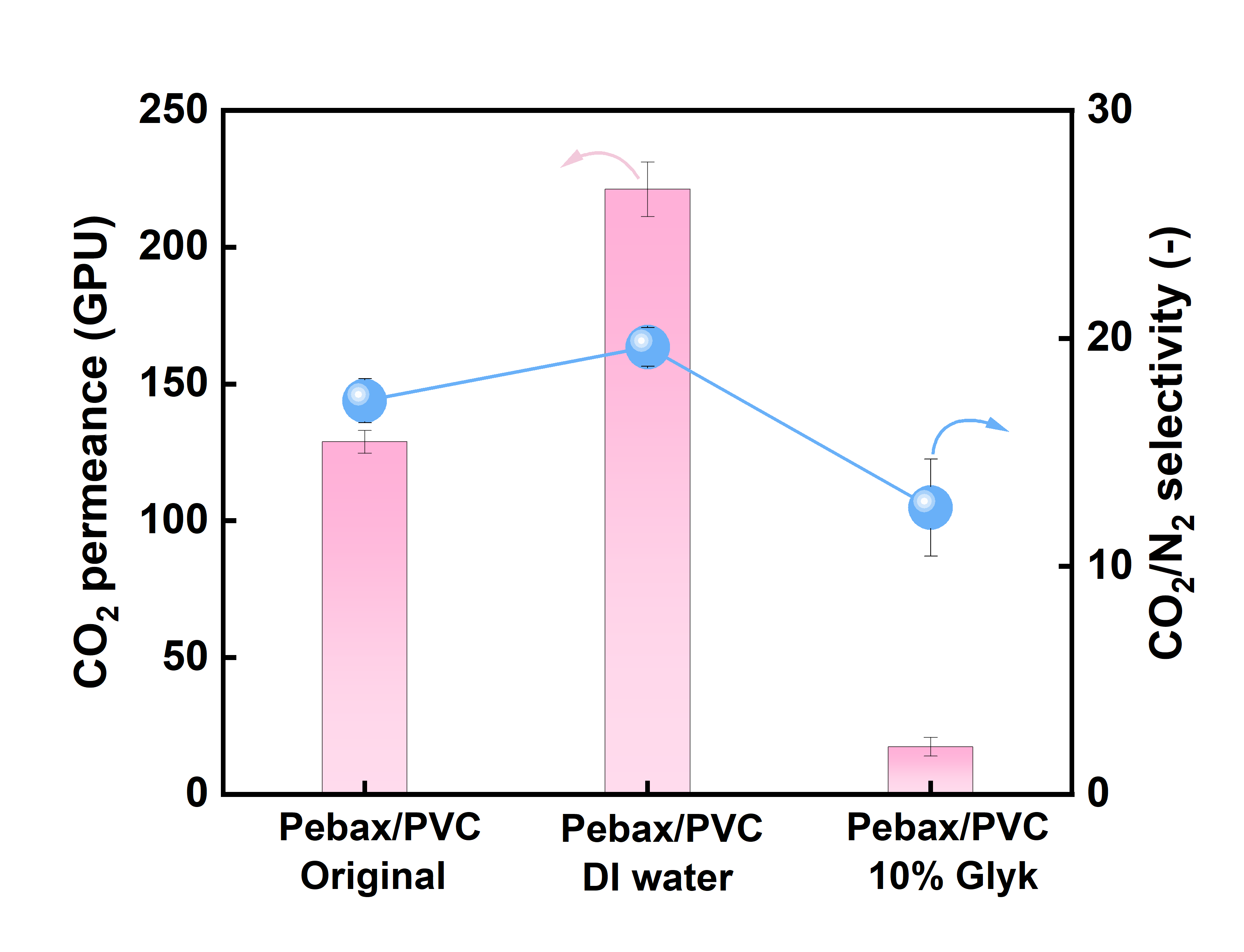


# Figure S21. Comparison of CO_2_ gas separation performance of HF membrane modules before and after MSR (mixed gas, 25 °C, 2 bar).


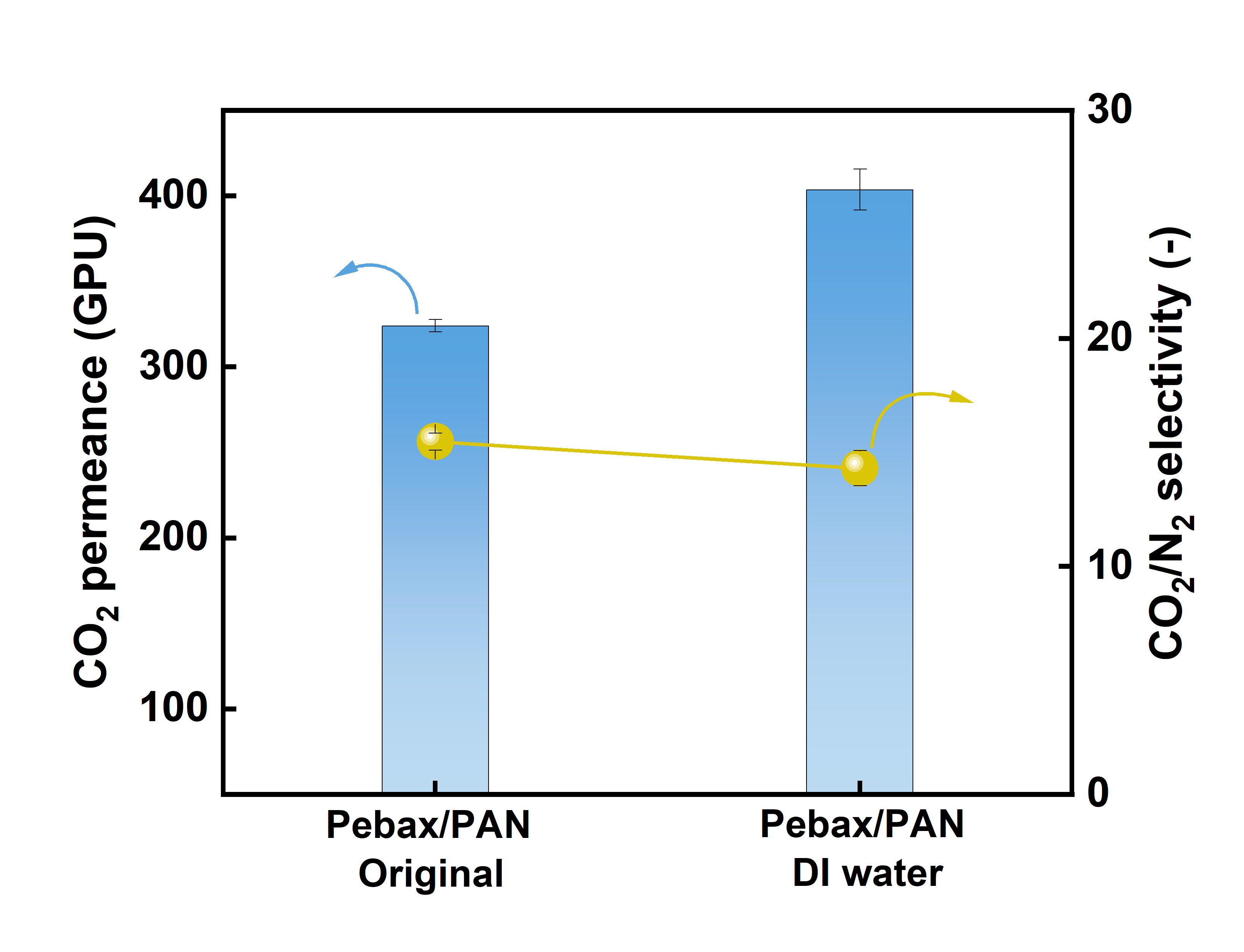


# Figure S22. CO_2_/N_2_ separation performance of HF membrane modules before and after MSR (mixed gas, 25 °C, 2 bar).


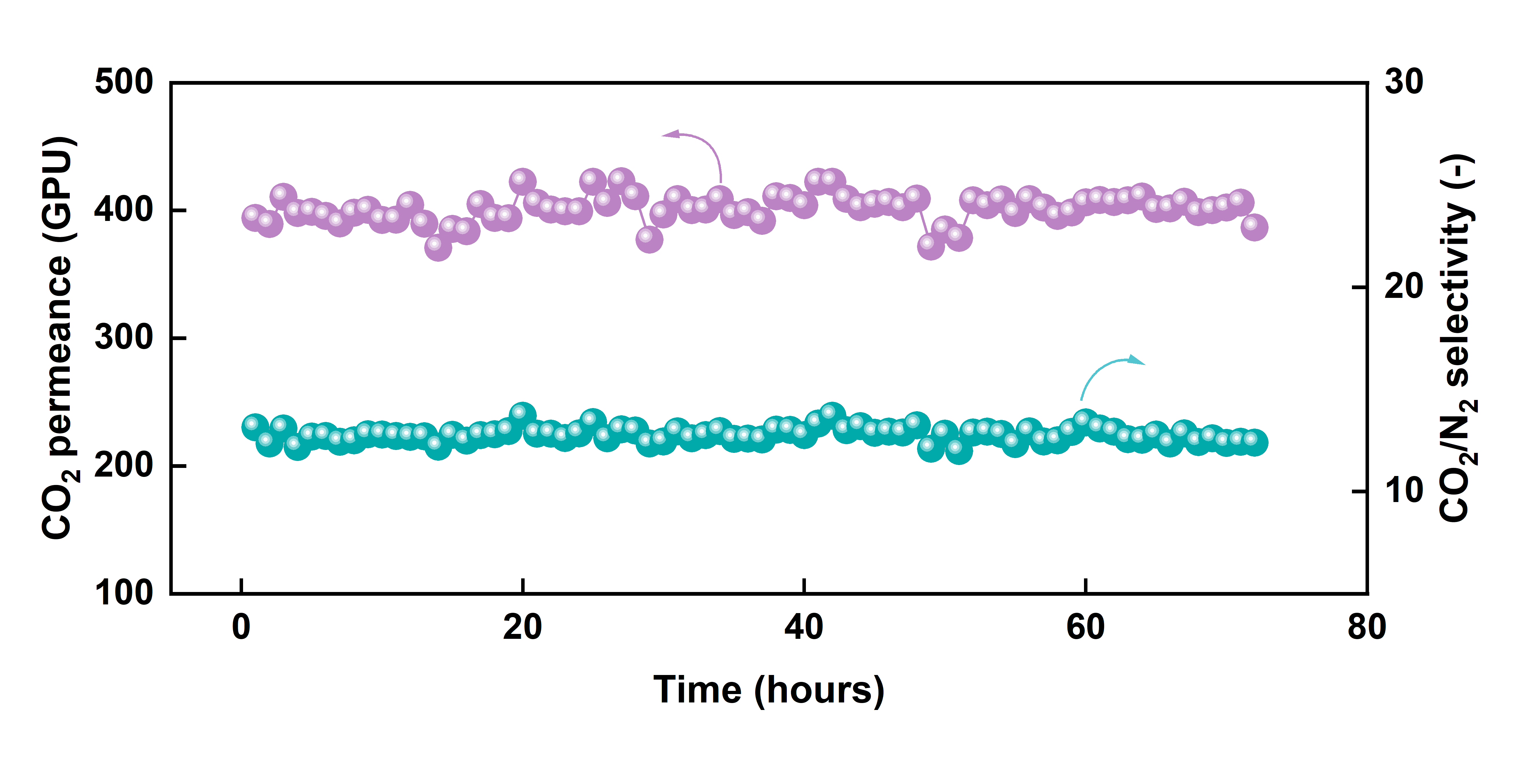


# Figure S23. Long-term stability testing of flat sheet Pebax^TM^/PAN TFC membranes (mixed gas, 25 °C, 2 bar).


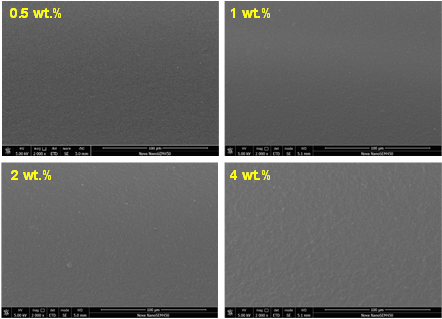


# Figure S24. SEM images of the HF TFC membranes surfaces with different casting solution concentrations.

# Reference

[1] R. Casadei, M. Giacinti Baschetti, B. G. Rerolle, H. B. Park, L. Giorgini, *Polymer* **2021**, *228*, 123944, https://doi.org/https://doi.org/10.1016/j.polymer.2021.123944.

[2] J. Wei, Y. M. Z. Qin, J. Deng, R. Selyanchyn, N. Li, L. Deng, S. Yi, R. Spontak, Z. Dai, *Polymer reviews*, **2025**, https://doi.org/https://doi.org/10.1080/15583724.2.

[3] B. Zhang, C. Yang, Y. Zheng, Y. Wu, C. Song, Q. Liu, Z. Wang, *Journal of Membrane Science*, **2021**, *627*, 119239, https://doi.org/https://doi.org/10.1016/j.memsci.2021.119239.

[4] B. Zhu, S. He, Y. Wu, S. Li, L. Shao, *Engineering*, **2022**, https://doi.org/https://doi.org/10.1016/j.eng.2022.03.016.

[5] E. Ahmadpour, A. A. Shamsabadi, R. M. Behbahani, M. Aghajani, A. J. Kargari, *Journal of Natural Gas Science Engineering*, **2014**, *21*, 518, https://doi.org/10.1016/j.jngse.2014.09.021.

[6] F. Dorosti, M. Omidkhah, R. J. Abedini, Design, *Chemical Engineering Research*, **2014**, *92* (11), 2439, https://doi.org/10.1016/j.cherd.2014.02.018.

[7] S. R. Reijerkerk, M. H. Knoef, K. Nijmeijer, M. J. Wessling, *Journal of membrane science*, **2010**, *352* (1-2), 126, <https://doi.org/10.1016/j.memsci.2010.02.008>.

[8] Z. Dai, H. Aboukeila, L. Ansaloni, J. Deng, M. Giacinti Baschetti, L. Deng, *Separation and Purification Technology,* **2019**, *214*, 67, https://doi.org/https://doi.org/10.1016/j.seppur.2018.03.062.

[9] T. Peters, L. Ansaloni, M. Rosa De La Viuda, A. Tena, O. Karvan, T. Visser, D. Chinn, N. Bhuwania, *Industrial & Engineering Chemistry Research*, **2025**, *64* (6), 3441, https://doi.org/10.1021/acs.iecr.4c03485.

[10] I. Taniguchi, S. Duan, T. Kai, S. Kazama, H. J. J. o. M. C. A. Jinnai, *Journal of Materials Chemistry A* **2013**, *1* (46), 14514. https://doi.org/10.1039/C3TA13711B

[11] W. Ji, K. Li, Y.-G. Min, W. Shi, J. Li, X. Ma, *Journal of Membrane Science* **2021**, *623*, 119091, https://doi.org/https://doi.org/10.1016/j.memsci.2021.119091

[12] G. S. Golubev, S. E. Sokolov, T. N. Rokhmanka, D. S. Bakhtin, I. L. Borisov, A. V. Volkov, *Membranes and Membrane Technologies* **2022**, *4* (6), 404, https://doi.org/10.1134/S2517751622060038.

[13] J. Gao, H. Mao, H. Jin, C. Chen, A. Feldhoff, Y. Li, *Microporous and Mesoporous Materials*, **2020**, *297*, 110030, https://doi.org/https://doi.org/10.1016/j.micromeso.2020.110030.

[14] A. Ehsani, M. Pakizeh, J. *Taiwan Inst. Chem. Eng*, **2016**, *66*, 414, https://doi.org/https://doi.org/10.1016/j.jtice.2016.07.005.

[15] M. M. Rahman, V. Filiz, S. Shishatskiy, C. Abetz, S. Neumann, S. Bolmer, M. M. Khan, V. J. Abetz, *Journal of Membrane Science*, **2013**, *437*, 286, https://doi.org/10.1016/j.memsci.2013.03.001.

[16] V. Nafisi, M.-B. Hägg, *Journal of Membrane Science*, **2014**, 459, 244, https://doi.org/https://doi.org/10.1016/j.memsci.2014.02.002.

[17] Y. Wu, D. Zhao, S. Chen, J. Ren, K. Hua, H. Li, M. Deng, *Separation and Purification Technology*, **2021**, *261*, 118243, https://doi.org/https://doi.org/10.1016/j.seppur.2020.118243.

[18] R. Casadei, M. Giacinti Baschetti, M. J. Yoo, H. B. Park, L. J. Giorgini, *Membranes*, **2020**, *10* (8), 188, https://doi.org/10.3390/membranes10080188.

[19] J. Deng, Z. Dai, L. Deng, *Industrial & Engineering Chemistry Research*, **2020**, *59* (32), 14458, https://doi.org/10.1021/acs.iecr.0c01946.

[20] H. Jiang, L. Bai, B. Yang, S. Zeng, H. Dong, X. Zhang, *Chinese Journal of Chemical Engineering*, **2022**, *43*, 169, https://doi.org/https://doi.org/10.1016/j.cjche.2022.02.006.

[21] P. Bernardo, J. C. Jansen, F. Bazzarelli, F. Tasselli, A. Fuoco, K. Friess, P. Izák, V. Jarmarová, M. Kačírková, G. Clarizia, *Separation and Purification Technology*, **2012**, *97*, 73, https://doi.org/https://doi.org/10.1016/j.seppur.2012.02.041.
